# MYC Modulation around the CDK2/p27/SKP2 Axis

**DOI:** 10.3390/genes8070174

**Published:** 2017-06-30

**Authors:** Per Hydbring, Alina Castell, Lars-Gunnar Larsson

**Affiliations:** 1 Department of Oncology-Pathology, Karolinska Institutet, SE-171 76 Stockholm, Sweden; per.hydbring@ki.se; 2 Department of Microbiology, Tumor and Cell Biology, Karolinska Institutet, SE-171 77 Stockholm, Sweden; alina.castell@ki.se

**Keywords:** cancer, oncogenes, tumor suppressor genes, cell cycle, cellular senescence, transcription, phosphorylation, post-translational modifications, the ubiquitin/proteasome system, protein–protein interactions

## Abstract

MYC is a pleiotropic transcription factor that controls a number of fundamental cellular processes required for the proliferation and survival of normal and malignant cells, including the cell cycle. MYC interacts with several central cell cycle regulators that control the balance between cell cycle progression and temporary or permanent cell cycle arrest (cellular senescence). Among these are the cyclin E/A/cyclin-dependent kinase 2 (CDK2) complexes, the CDK inhibitor p27^KIP1^ (p27) and the E3 ubiquitin ligase component S-phase kinase-associated protein 2 (SKP2), which control each other by forming a triangular network. MYC is engaged in bidirectional crosstalk with each of these players; while MYC regulates their expression and/or activity, these factors in turn modulate MYC through protein interactions and post-translational modifications including phosphorylation and ubiquitylation, impacting on MYC’s transcriptional output on genes involved in cell cycle progression and senescence. Here we elaborate on these network interactions with MYC and their impact on transcription, cell cycle, replication and stress signaling, and on the role of other players interconnected to this network, such as CDK1, the retinoblastoma protein (pRB), protein phosphatase 2A (PP2A), the F-box proteins FBXW7 and FBXO28, the RAS oncoprotein and the ubiquitin/proteasome system. Finally, we describe how the MYC/CDK2/p27/SKP2 axis impacts on tumor development and discuss possible ways to interfere therapeutically with this system to improve cancer treatment.

## 1. Introduction

**Figure 1:**
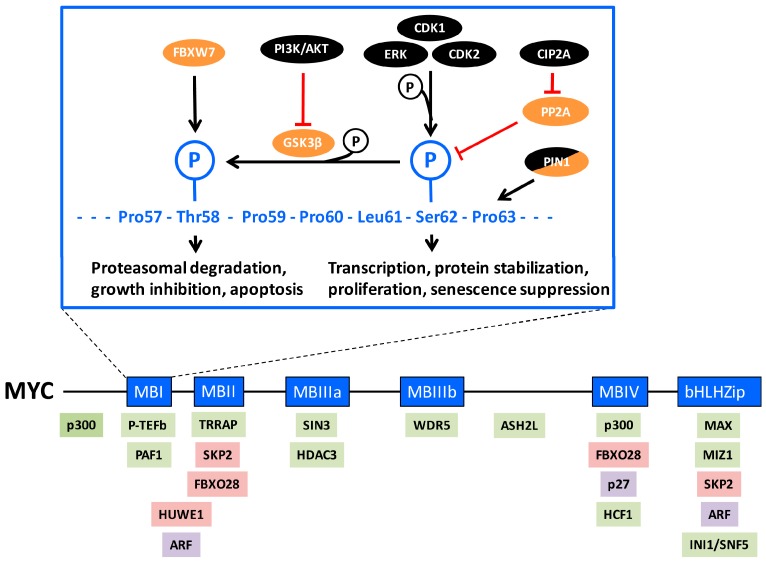
MYC structure and interaction partners. Lower part: The structure of MYC with evolutionary conserved regions of MYC, including the MYC boxes (MB) 1–4 and basic region/helix-loop-helix/leucine zipper (bHLHZip) domain, are depicted as blue boxes. Proteins of interest for this review interacting with respective regions, including regulators of transcription/chromatin, components of E3 ubiquitin ligase complexes and proteins with other functions described in the text are indicated in light green, pink or violet, respectively. Upper part: Enlargement of MB1. Proteins involved in regulation of the phosphorylation status of threonine-58 (Thr-58) and serine-62 (Ser-62), and the biological output of these phosphorylations are indicated. Ser-62, which plays a role in regulating protein stability, transcription, proliferation and oncogenesis, is phosphorylated by indicated kinases. This phosphorylation facilitates glycogen synthase kinase 3β (GSK3β)-mediated phosphorylation of Thr-58, which is a signal for F-box protein FBX7-mediated proteasomal degradation linked to growth suppression and apoptosis. The activity of GSK3β is blocked by phosphorylation via the phosphatidylinositol-3-kinase (PI3K)/AKT pathway. The phosphorylation status of Ser-62 is also regulated by the protein phosphatase 2A (PP2A) with assistance from the PIN1 prolyl isomerase acting on proline-53 (Pro-53). PP2A can be blocked by endogenous inhibitory proteins such as cancerous inhibitor of PP2A (CIP2A). Proteins, depicted in black, represent factors with growth-promoting/oncogenic function, while those in orange represent growth/tumor-suppressive function in this context. PIN1, which plays a dual function, is depicted partly in black and partly orange. See the text for further explanation.

The MYC oncogene family, consisting of *MYC*, *MYCN*, and *MYCL*, here collectively referred to as “MYC”, encodes transcription factors that contain a basic region/helix-loop-helix/leucine zipper (bHLHZip) type of DNA-binding and protein interaction domain. All MYC proteins heterodimerize with the obligatory bHLHZip protein MAX, which enables the MYC:MAX heterodimer to bind so-called E-box DNA sequences (CACGTG and similar sequences) situated in regulatory regions of target genes [1,2,3,4,5]. In doing so, MYC has the ability to regulate target genes by recruiting different cofactors participating in chromatin modification and remodeling and/or in the initiation and elongation of RNA Pol I, II, and III-mediated transcription. This involves protein–protein interactions with a number of proteins, engaging the evolutionary conserved MYC boxes (MB) 1–4 situated in the transactivation domain (TAD) and other parts of the MYC protein [5,6] (Figure 1, lower part). Examples of such cofactors are TRRAP, which binds MYC via MYC box 2 (MB2) and is part of the SAGA and other histone acetyl transferase (HAT) complexes [7]. MYC also binds the HAT-containing cofactor p300/CBP [8,9,10,11,12]. Post-translational modifications such as acetylation, methylation, phosphorylation and ubiquitylation at specific sites along histone tails play important roles in regulation of chromatin structure and transcription [13]. Recently two components of the multisubunit COMPASS/mixed lineage leukemia (MLL) histone H4 methylase complexes, WRD5 [14] and ASH2L [15], were found to interact with MYC, the former which seems to stabilize MYC’s interaction with chromatin [14], while interaction with the latter was shown to promote histone H3 lysine 27 (H3K27) demethylation and subsequent H3K27 acetylation [15]. H3K27 acetylation is like histone H3 lysine 4 (H3K4) methylation a mark for actively transcribed genes [16]. Further, the INI1/SNF5 subunit of ATP-dependent chromatin remodeling complexes has been reported to interact with the bHLHZip domain of MYC. The area around MYC box 1 (MB1), which like MB2 is part of the TAD, plays a regulatory role and interacts with a number of factors controlling MYC activity and turnover (see further below), as well as positive transcription elongation factor P-TEFb (cyclin T/CDK9) [17,18,19] and PAF1 [20], which play important roles in regulation of transcription elongation. In addition, MYC also interacts with other specific DNA-binding transcription factors including MIZ1, SP1 and SMADS [21,22,23]. The former takes part in MYC-mediated repression of transcription [24,25,26,27]. Repression is also mediated by MYC’s binding to SIN3 [28] and HDAC3 [29], which are part of histone deacetylase (HDAC) transcriptional repressor complexes. Through these protein–DNA and protein–protein interactions, MYC directly regulates transcription of a large number of genes, which in turn affects global transcription and protein synthesis, thereby controlling a number of fundamental processes in the cell including cell growth and division, cell survival and differentiation, metabolism, cellular senescence and stem cell functions [2,3,4,5,26,30,31,32,33]. 

The *MYC* oncogenes are key players in cancer initiation and progression, being critical for maintaining the tumorigenic state in numerous cancer types. Since they function as such a broad-ranged transcription factor, levels of MYC availability and magnitude of MYC activity need to be in tight control to balance the cell’s transcriptional output. This tight control is lost during tumor development. In contrast to many other oncogenes, the oncogenic features of the MYC family genes and gene products are mainly characterized by deregulated gene expression, caused by alterations at the DNA, RNA and/or protein levels through for instance gene amplification, chromosomal translocation, transcriptional deregulation or protein stabilization [2,3,4,5]. In fact, the MYC locus is the most frequently amplified locus across human tumors, leading to MYC overexpression [34]. Regulation of transcription of the MYC locus is very complex and is under the control of a number of different proximal promoter elements and distal enhancers, including super-enhancers, that are able to respond by numerous signaling pathways involved in cell proliferation, survival, differentiation or other cellular cues [2,5,30,35,36,37,38,39]. Deregulation of such signaling pathways in cancer therefore frequently leads to deregulation of MYC expression. Further, the MYC protein is subjected to a number of modifications that regulate MYC activity and/or turnover, including phosphorylation, ubiquitylation (proteolytic or non-proteolytic), acetylation and small ubiquitin-related modifier (SUMO)-ylation [3,4,40,41,42,43,44]. Cellular signal transduction pathways in turn regulate the activities of the enzymes responsible for these modifications. Regulation of MYC protein level occurs mainly by the ubiquitin/proteasome system and is also responsive to cell signaling. The MYC protein is very short-lived, having a half-life of around 30 min. A number of E3 ubiquitin ligases have been implicated in ubiquitylation of MYC, including SKP2, FBXW7, HUWE1/HECTH9, FBX29, βTRCP, TRUSS, PIRH2, FBXO28, CHIP and FBXL14 (for review see [40,44]). In addition, MYC protein levels are regulated at the level of mRNA translation through the 5′ cap mRNA-binding eIF4F complex but also through internal ribosomal entry site elements that are bound by eIF4A-containing complexes, both of which are controlled by mammalian target of rapamycin (mTOR) signaling [45,46,47,48].

This review focuses on regulation of MYC through protein–protein interactions and post-translational modifications at MYC-boxes 1 and 4 involving cyclin-dependent kinase 2 (CDK2), the cyclin-dependent kinase inhibitor p27^KIP1^ (referred to from now on as p27) and the S-phase kinase-associated protein 2 (SKP2), together constituting the CDK2/p27/SKP2 network, and the impact this pathway has on MYC-regulated transcription and tumorigenesis. We further elaborate on possible therapeutic interventions within this pathway as a potential new strategy to target MYC in cancer.

## 2. MYC Regulation by Phosphorylations in MYC-Box 1

The N-terminal MYC-box 1 (MB1) in the transactivation domain is one of the important regulatory regions of MYC, playing a role in transcriptional activity and transformation. It also encompasses a conserved phospho-degron, i.e., an amino acid sequence containing a phosphorylation site(s) involved in protein degradation [40,49]. Two phosphorylation sites in particular, threonine 58 (Thr-58) and serine 62 (Ser-62), are essential for this regulation (Figure 1, upper part). Ser-62 is phosphorylated by ERK and other mitogen-activated protein kinases (MAPK) as a result of mitogenic RAS or stress signaling [50,51,52,53,54,55,56] but also by cyclin-dependent kinases CDK2, CDK1 and CDK5 [54,57,58,59,60]. Further, the PIM1 kinase has been reported to be engaged in this phosphorylation [61,62], but it is unclear if this effect is direct or indirect. Phosphorylation at Ser-62 facilitates (“primes”) subsequent phosphorylation at the nearby Thr-58 site by glycogen synthase kinase 3β (GSK3β) [51,53,63]. The phosphorylated Thr-58/Ser-62 residues constitute a binding site for the F-box protein FBXW7 [64,65], which is the substrate binding subunit of a larger so-called SCF (Skp1–Cullin1–F-box) E3 ubiquitin ligase complex [66]. This results in poly-ubiquitylation and the subsequent degradation of MYC by the 26S proteasome. Mutation of Thr-58 occurs in about half of Burkitt’s lymphomas, leading to stabilization of MYC due to impaired proteasome turnover and evasion of apoptosis [53,67,68,69,70]. Loss of FBXW7 results in decreased turnover of MYC for the same reason, and has been reported in uterine, colorectal and bladder cancers, T-acute lymphoblastic leukemia (ALL) among other tumors [34,71,72]. Since GSK3β is negatively regulated by AKT phosphorylation [73], activation of the phosphatidylinositol-3-kinase (PI3K)/AKT pathway, for instance through RAS signaling can lead to stabilization of Ser-62-phosphorylated MYC by preventing GSK3β-mediated Thr-58 phosphorylation [53,74,75,76].

Adding another layer of complexity, Sears’ laboratory reported that degradation of MYC required PIN1-assisted de-phosphorylation at Ser-62 by protein phosphatase 2A (PP2A) [56]. According to this model, prolyl isomerization of proline 63 (Pro 63) from *cis* to *trans* by PIN1 in Thr-58/Ser-62 double phosphorylated MYC enables dephosphorylation of Ser-62 by PP2A, and subsequent FBXW7-mediated MYC degradation (Figure 1, upper part). In this process, Axin acts as a scaffold, bringing together phosphorylated MYC, GSK3β, PIN1, PP2A and FBXW7 [77]. Splice mutations in Axin affecting its scaffolding function occur in some cancers, thereby impairing MYC degradation [78]. Furthermore, Westermarck’s laboratory reported that de-phosphorylation of Ser-62 by PP2A was counteracted by the protein Cancerous Inhibitor of PP2A (CIP2A), leading to MYC stabilization [79]. CIP2A has been found to be overexpressed in many types of cancers [80].

It is, however, still unclear why de-phosphorylation of Ser-62 would be required for FBXW7-mediated degradation, since FBXW7 binds a double Ser-62/Thr-58 phosphorylated MYC peptide with as high or higher affinity [65], and since FBXW7 binds and ubiquitylates many other substrates in a double phosphorylated configuration similar to Ser-62/Thr-58 in MB1 [72]. Possibly, phosphorylated Ser-62 provides a binding site in the cell for another protein(s) stabilizing MYC, perhaps by excluding binding by FBXW7. This remains to be elucidated.

## 3. Role of Ser-62 Phosphorylation in the Regulation of MYC’s Biological Activity

Although still somewhat of a conundrum, evidence is accumulating that phosphorylation at Ser-62 not only primes for Thr-58 phosphorylation and MYC destruction, but also plays an independent role in MYC regulation (Figure 1, upper part). If the sole function of phosphorylated Ser-62 would be to stimulate Thr-58 phosphorylation, one would expect Ser-62 missense mutations to impact MYC in a similar way as Thr-58 mutations, namely stabilization of MYC and increased transforming capacity. However, it was reported early on that transformation of primary rat embryo fibroblasts (REFs) by RAS and MYC, was potentiated by MYC T58A but severely reduced by a S62A mutant [81,82], although this phenomenon may be linked to activated RAS signaling since it was not observed in the absence of mutant RAS [83]. Similar results were obtained in primary human fibroblasts transformed by RAS, MYC and telomerase (hTERT); HEK cells expressing RAS, hTERT and T58A MYC formed tumors in vivo, while RAS and hTERT in combination with S62A did not [56]. Using the primary REF system, our laboratory showed that wild-type (WT) MYC and T58A MYC suppressed RAS-induced cellular senescence, while the S62A mutant was unable to do so [58]. Taken together, these results suggest that the Thr-58 and Ser-62 phosphorylations support two independent, opposite functions, at least with respect to cooperation with RAS, and that mutation of the Ser-62 phosphorylation site interferes with MYC and RAS cooperativity during transformation (Figure 1, upper part). Further information about the biological role of Ser-62 comes from Sears’ laboratory, which utilized MYC knock-in mice where either WT MYC or MYC phosphorylation mutants were specifically expressed in mammary glands [84]. WT MYC increased mammary gland density but did not induce tumors. Interestingly, the MYC Ser-62 mutant mice displayed decreased mammary gland density and ductal branching compared to WT mice. Intriguingly, both MYC Ser-62 and Thr-58 mutant mice displayed genomic instability, but only MYC Thr-58 mutant mice developed carcinomas. This difference could be attributed to the onset, or lack thereof, of intrinsic tumor suppressor mechanisms. Indeed, expression of the Thr-58 mutant in the mammary gland suppressed apoptosis [84], and possibly also senescence, as reported in fibroblasts [58], while expression of the Ser-62 mutant did not. 

## 4. Role of Ser-62 Phosphorylation in Stabilization of MYC

Why would Ser-62 phosphorylation increase the biological activity of MYC? One suggested mechanism is protein stabilization (Figure 1, upper part) [53], supported also by the observed de-phosphorylation of Ser-62 by PP2A during FBXW7-mediated ubiquitylation and degradation [56]. Serum stimulation or ectopic expression of RAS (which both induce Ser-62 phosphorylation) was reported to stabilize WT MYC but not the S62A mutant [53]. Further, inhibition of PP2A, which increases Ser-62 phosphorylation, stabilized MYC, while depletion of the PP2A inhibitory protein CIP2A, which decreases Ser-62 phosphorylation, destabilized MYC [56,79], thus demonstrating a good correlation between Ser-62 phosphorylation and MYC stability. CIP2A was also shown to be required for the induced MYC stability after serum stimulation [85]. 

Contradictory to these reports, CDK1-induced phosphorylation of MYCN at Ser 54 (which corresponds to MYC Ser-62 in the evolutionary conserved MB1) did not stabilize the protein during mitosis in neuronal cells, but instead resulted in rapid degradation of MYCN via GSK3β-mediated phosphorylation of Thr-50 (corresponding to MYC Thr-58). Further, inhibition of PP2A also increased degradation of MYC in this system, correlating with increased Ser-62 phosphorylation [60]. 

Although there seems to be a general agreement that Thr-58 phosphorylation destabilizes MYC, there are thus conflicting data on whether Ser-62 phosphorylation affects MYC stability. If Ser-62 phosphorylation stabilizes MYC, one would expect a S62A mutant to decrease stability. However, this mutant has been reported to exhibit a similar half-life to the WT MYC in REF52 cells [53], or increased stability such as in primary cerebellar granule neuron precursor cells [74] as well as in 2fTGH fibrosarcoma cells [58] compared to WT MYCN and WT MYC, respectively. Further, expression of this mutant in the mouse mammary gland suggested that it has increased stability compared to WT MYC [84,85]. 

What could be the explanation for these discrepancies? The literature seems to agree that as a priming event, Ser-62 phosphorylation does promote FBXW7-induced MYC degradation. However, the execution of this signal can be enhanced/speeded up or dampened/delayed by a number of other players, including PP2A, PIN1 and GSK3β in response to RAS, PI3K/AKT or WNT signaling, as mentioned above. In addition, FBXW7-induced degradation of MYC is fine-tuned by the de-ubiquitylation enzyme USP28 [86], which slows down poly-ubiquitylation, as well as by the E3 ligase βTRCP, which counteracts FBXW7 by conjugating mixed Ub chains to MYC that disfavors degradation. All these players will be differentially expressed or active in various tissues and phases of the cell cycle depending on proliferative potential or tumorigenic status. Under certain conditions, a “window of opportunity” may be created where Ser-62 phosphorylation transiently stabilizes MYC. Furthermore, one should remember that Ser-62 mutants might not be equivalent to unphosphorylated Ser-62. Destabilization may require an intact serine at position 62, potentially forming a binding site for a destabilizing factor or a site for a competing non-phosphate conjugation such as glycosylation [87]. The regulation of MYC stability by PP2A is further complicated by the finding that PP2A dephosphorylates an inhibitory phosphorylation site on GSK3β, thereby promoting Thr-58 phosphorylation [88]. This finding provides an alternative or additional explanation why PP2A inhibition stabilizes MYC. It is also important to remember that the overall speed of degradation of MYC in a certain situation will be the sum of the activity of a number of different MYC-targeting E3-ligases (see Introduction), many of which are responsive to signaling. 

Taken together, although stabilization of MYC by Ser-62 phosphorylation may contribute to the increased potency of MYC in the biological systems described above, this is unlikely to be the sole explanation. 

## 5. Role of Ser-62 Phosphorylation in MYC-Regulated Transcription

Experiments performed already in the early 90s using the MYC TAD fused to the DNA binding domain of GAL4 in promoter/reporter assays suggested that the activity of the MYC TAD was enhanced by serum stimulation or by ERK2, and was regulated during the cell cycle in a Ser-62 dependent manner [54,89]. Similar transient promoter/reporter experiments using full-length WT MYC or phospho-mutants acting on E-box-driven reporters have given conflicting results, from reduced to unaltered activity comparing WT MYC and a S62A mutant [51,90]. One explanation for the discrepancies could be that these experiments were carried out in exponentially growing unstimulated cells. Studying regulation of endogenous MYC-target genes, Watnik et al. [55] found that RAS-induced phosphorylation of Ser-62 via p38MAPK was required for repression of the *thrombospondin* (*TSP*-1) gene, a regulator of angiogenes, as well as activation of the well-known MYC target gene *ornithine decarboxylase* 1 (*ODC*1). Expression of the MYC S62A mutant reversed these changes in *TSP*-1 and *ODC*1 expression, and apparently behaved as a dominant negative mutant in this setting.

In what way could Ser-62 phosphorylation affect MYC-regulated transcription mechanistically? Benassi et al. [50] found that ERK-mediated phosphorylation of Ser-62 in response to oxidative stress resulted in recruitment of MYC to the promoter of the γ-*glutamyl-cysteine synthetase* (γ-*GCS*) gene—a key enzyme in glutathione synthesis. Although expressed at the same level as WT MYC, MYC S62A was not recruited to the promoter, and expression of this mutant increased oxidative stress-induced cell death. Our laboratory showed that MYC suppressed RAS-induced cellular senescence in REFs in a Ser-62-dependent but Thr-58-independent manner. Pharmacological inhibition of CDK2-mediated Ser-62 phosphorylation resulted in depletion of MYC from genes involved in senescence regulation despite continuous MYC expression, resulting in abrogation of MYC-mediated senescence suppression. These studies suggest that Ser-62 phosphorylation is involved in recruitment and maintenance of MYC at subsets of MYC-regulated genes involved in managing oncogene-induced and other types of stresses that can cause apoptosis or senescence [50,58]. 

The question remains how phosphorylation of Ser-62 affects the association of MYC with certain genomic loci and MYC-dependent gene regulation? Sears’ laboratory recently reported that the prolyl isomerase PIN1 increases the rate of recruitment of MYC to chromatin [91]. This required the phospho-binding and isomerase domains of PIN1 and phosphorylated MYC Ser-62, suggesting that PIN1 activity is involved at two steps in MYC regulation; firstly, by facilitating MYC recruitment to DNA and secondly by stimulating Thr-58/FBXW7-mediated turnover, both in a Ser-62-dependent manner. In addition, the authors demonstrated that PIN1 enhanced recruitment of cofactors involved in MYC-driven transcription to select promoters, including the HAT components p300 and GCN5, the P-TEFb subunit CDK9 and the INI1/SNF5 chromatin remodeling complex subunit. This correlated with increase histone acetylation and RNA polymerase II (pol II) presence at gene bodies, indicative of enhanced transcription elongation [91]. It is unclear from this study whether the recruitment of these cofactors is a consequence of PIN1’s effect on MYC DNA binding, or whether PIN1 primarily stimulates binding of these cofactors to MYC, which in turn leads to enhanced recruitment and affinity of MYC to chromatin, or both. It also remains to be clarified how PIN1-mediated isomerization through Ser-62 contributes to MYC and cofactor recruitment to chromatin.

Evidence is emerging that both the phosphorylated and unphosphorylated Ser-62 residue may act as binding sites for different factors. The tumor suppressor protein BIN1 was shown to interact with unphosphorylated Ser-62, but displaced upon Ser-62 phosphorylation [92]. Another study suggested that BIN1 inhibits transactivation of target genes by MYC and to mediate MYC-induced apoptosis [93,94], but it is still unclear how this works mechanistically. Recently, Jaenicke et al. [20] demonstrated that a peptide containing MB1 phosphorylated at Thr-58 and Ser-62 was able to interact with PAF1C, which is a transcription elongation factor facilitating RNA Pol II transcription through nucleosomal barriers [95]. This is consistent with findings that MYC promotes transcription elongation [17,96,97]. In addition, the PP2A inhibitor CIP2A mentioned above was reported to bind MYC in a Ser-62-dependent manner, thereby protecting Ser-62 from de-phosphorylation by PP2A [79]. Recently, Myant et al. [85] suggested that the interaction between Ser-62-phosphorylated MYC and CIP2A takes place at the nuclear lamina, where nuclear CIP2A is enriched, whereas the S62A mutant associated with these structures to a much lesser extent. According to Myant et al.’s model, Ser-62-phosphoryled MYC is recruited to lamin A/C-associated nuclear structures (LAS) by CIP2A, where it regulates genes localized to the LAS. The LAS is known to be enriched in heterochromatin, i.e., densely packed chromatin associated with gene silencing, but the extent of gene silencing/activation may depend on the local chromatin environment and regulatory sequences [98]. The authors further used a mouse model for intestinal regeneration after DNA damage, which requires MYC function [99,100], and found that depletion of CIP2A inhibited regeneration [85]. In accordance with the in vitro data, loss of Ser-62-phosphorylated MYC from the nuclear lamina and concomitant reduced expression of MYC target genes was observed in regenerating intestines of CIP2A deficient mice. Further, replacing the endogenous MYC allele with the S62A mutant led to reduced ability to rescue intestinal regeneration compared to the T58A mutant, despite similar expression levels.

Taken together these reports suggest that phospho-Ser-62-interacting proteins influence activity, target gene selection and intracellular localization of MYC (Figure 1, upper part).

Another important question is whether the transcriptional output of Ser-62 phosphorylated MYC is dictated by which kinase executes this phosphorylation. As mentioned above, suppression of RAS-induced cellular senescence by MYC involved CDK2-mediated phosphorylation of Ser-62 [58]. Surprisingly, while pharmacological inhibition of CDK2 resulted in reestablishment of RAS-induced senescence, inhibition of ERK or CDK1 had no impact on this process [58], arguing for a unique role of CDK2 in modulating MYC-regulated senescence. This may suggest that the different Ser-62 kinases play different roles in MYC regulation, possibly by also phosphorylating additional sites on MYC, which has been proposed for JNK, CDK2 and CDK5 [52,60] and that may influence the outcome. Other possibilities are that different Ser-62 kinases simultaneously and selectively phosphorylate other relevant substrates or carry out kinase-independent functions specific for each kinase, all of which may have an impact on MYC regulation and function. 

To fully understand to role of Ser-62 phosphorylation, the repertoire of MYC target genes sensitive to this modification and the collection of MYC-interacting factors involved in this regulation need to be characterized in various tissues and in response to different signaling, for instance by genome-wide chromatin immunoprecipitation (ChIP) and RNA-sequencing and proteomics studies. Further, mice expressing phosphorylation mutants of MYC, some of which were exemplified above, could be depleted of Ser-62 kinase candidates by any of the currently available technologies (crosses with transgenic, strains, CRISPR/CAS9 or RNA interference (RNAi) techniques, etc.) to fully decipher the role of Ser-62 phosphorylation in regulating MYC transactivation activity and stability. 

In the next sections we put particular focus on the role of CDK2, not only as a Ser-62 kinase but also with respect to its other substrates and interaction partners including p27 and SKP2, in MYC regulation. 

## 6. The Crosstalk between MYC and CDK2

**Figure 2:**
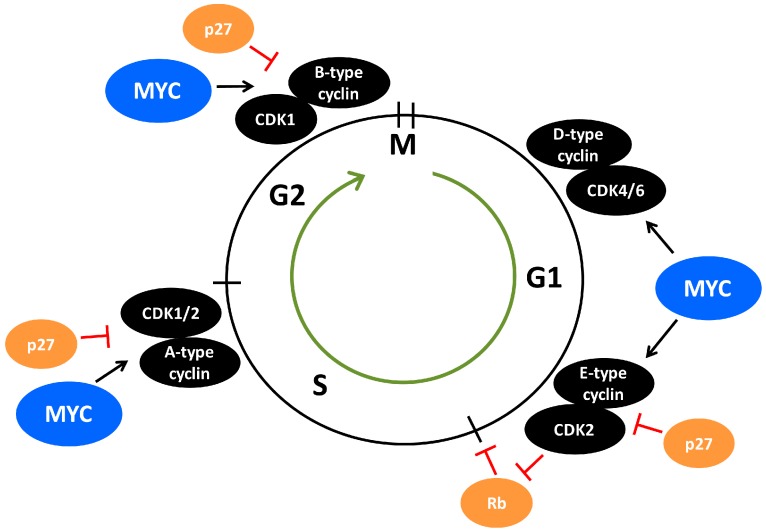
Experiments performed in the early 1990s using the MYC transactivation domain (TAD) fused to the DNA binding domain of GAL4 in promoter/reporter assays suggested that the activity of the MYC TAD was enhanced by serum stimulation or by ERK2, and was regulated during the cell cycle in a Ser-62 dependent manner [54,89]. The mammalian cell cycle. The cell cycle phases, gap 1 (G1), DNA synthesis (S), gap 2 (G2) and mitosis (M) and the different cyclin/cyclin-dependent kinase (CDK) complexes and their periods of activity during the cell are illustrated. The retinoblastoma protein (pRB), which blocks entry into S phase through transcriptional repression of E2F family transcription factors, is inactivated by D-type/E-type-cyclin/CDK2/4/6 phosphorylation thus enabling G1-S phase transition. The activities of the CDK2 and CDK1 complexes are inhibited by the CDK inhibitor p27^KIP1^ (p27). The points of intervention by MYC in the cell cycle are depicted. Proteins depicted in black represent factors with growth-promoting/oncogenic function (with the exception of MYC, which is colored blue), while those in orange represent growth/tumor-suppressive function in this context.

CDK2 is a core cell cycle component that is mainly active from late G1-phase and throughout the S-phase due to its activity being dictated by E-type and A-type cyclins fluctuating in their expression during the cell cycle (Figure 2). Growth factor signaling in G1-phase activates MYC, which stimulates cyclin E/CDK2 activity [101,102,103,104,105], in part by activating expression of cyclin D2 and CDK4 [106,107,108] (Figure 2). Cyclin D/CDK4 complexes contribute to CDK2 activation by sequestering the CDK2-inhibitor p27 [106,108]. In addition, MYC induces expression of components of the E3 ubiquitin ligase complex that targets p27 for proteasome-mediated turnover [109,110,111]. Buildup in cyclin E/CDK2 activity due to inhibition of p27 and increased cyclin E expression and stability finally force cells to transit into S-phase through phosphorylation of various targets [112,113,114] (Figure 2). Among the most important CDK2 substrates during G1-S progression are the RB-family of pocket proteins including the retinoblastoma protein (pRB) which is phosphorylated by cyclin E/CDK2 in cooperation with cyclin D/CDK4/6. This results in relieved transcription repression of S-phase genes by pRB in complex with E2F family transcription factors, leading to entry into S-phase [101,115,116,117,118,119,120,121]. There are numerous substrates reported for CDK2-cyclin E/A in addition to the RB-family of pocket proteins, some of which may represent functions distinct from cell cycle progression. Overall, many substrates are DNA-binding transcription factors, such as SMAD3, FOXM1, FOXO1, ID2, NFY, B-MYB and MYC [101], whereby CDK2 affects transcriptional output. Also other types of CDK2 substrates are reported including p27 [122,123,124] and the E3 ubiquitin ligase FBXO28 [9], both involved in MYC regulation [9,58,125] (see further below), the polycomb repressor protein EZH2 and the anti-apoptotic protein MCL1 [101]. 

Progression and completion of S-phase is regulated by A-type cyclins, which in complex with CDK2 promotes DNA replication and have functions in DNA damage repair [101,126] (Figure 2). Cyclin E/A-CDK2 has been reported to phosphorylate substrates directly involved in DNA replication, including CDC6 and multiple MCM helicase proteins, which are part of the pre-replicative complex that builds up at origins of replication during the initial step of DNA replication [127,128,129,130]. In support of this, cyclin E-null cells failed to load MCM helicase into the pre-replication complex [131]. Part of the stimulatory effect of cyclin E/CDK2 on replication seems also to be stimulation of the expression of components of the pre-replication complex [132]. Interestingly, MYC has also been reported to interact with these pre-replicative complex components and to regulate DNA replication independent of its role in transcription [133]. 

Our research laboratory and the laboratory of Bruno Amati demonstrated a direct connection between MYC and CDK2 in modulating cellular senescence in normal cells and cancer cells [58,134]. Mouse embryo fibroblasts (MEFs) devoid of CDK2 showed similar initial proliferation rates compared to WT cells in response to MYC-activation, but displayed premature onset of senescence [134]. Inhibition of CDK2 using pharmacological inhibitors in MEF WT cells with activated MYC also lead to a pronounced senescence response [134], as did blocking CDK2 activity in rat embryo fibroblasts co-expressing MYC and oncogenic RAS [58]. Genetic depletion of CDK2 (CDK2-/-) significantly delayed MYC-driven lymphomagenesis in mice and induced senescence, while induction of senescence was not observed in WT CDK2 mice, in concordance with the observations from cultured fibroblasts [134]. Induction of senescence in MYC-activated CDK2-/- MEFs could be bypassed by caffeine and other conditions impairing DNA damage response (DDR) signaling, suggesting that CDK2 is involved in adaption to MYC-induced cellular stress. Deregulated expression/activity of MYC has been shown to induce DNA damage through increased production of reactive oxygen species (ROS) due to excessive metabolic activity and/or through increased replication stress caused by abrogation of cell cycle checkpoints leading to overstimulation of replication, ultimately resulting in genomic instability, chromosomal aberrations and aneuploidy [133,134,135,136,137,138,139,140]. CDK2 has been linked to DNA damage repair by multiple studies [141,142,143,144,145,146,147]. Mechanistically, CDK2 has been proposed to control resection of DNA double-strand breaks by phosphorylating C-terminal binding protein (CTBP)-interacting protein (CtIP), thereby enhancing CtIP interaction with the homologous repair protein BRCA1 and the exonuclease MRE11 [142,145,146,147]. Unlike the WRN protein, which is required to avoid MYC-induced replication stress [139], CDK2 rather seems to be involved in abrogating oxidative stress, as supported by Amati and colleagues’ observation that MYC-induced senescence in CDK2-/- MEFs could be rescued by antioxidants [134]. Onset of senescence is characterized by a substantial increase in oxidative stress [134], a feature observed also in other systems of oncogene-induced senescence as well as during senescence triggered by cell culture shock [148,149,150]. The work from Amati’s laboratory therefore suggested that such damage is kept in check by CDK2.

Further, our laboratory demonstrated that MYC is directly targeted by CDK2-mediated phosphorylation at Ser-62, an event counteracted by pharmacological inhibitors of CDK2 and by overexpression of p27. As mentioned above, MYC is phosphorylated at Ser-62 in response to oxidative stress, which was shown to be required for MYC to balance this stress [50], thus further connecting these two events. We showed that MYC/CDK2 interactions localized to promoters, driving MYC target genes of specific importance for senescence outcome, including *hTERT*, *BMI-1*, *p16^INK4A^ and p21^CIP1^* [58]. Further, the expression of these genes was sensitive to CDK2 activity, as demonstrated by genetic or pharmacological depletion/inhibition of CDK2 or by expression of p27 [58,134]. Phosphorylation of Ser-62 by CDK2 could therefore be part of an MYC adaption program limiting oncogene-induced stress and suppressing senescence. Interestingly, although MYC is known to induce DNA damage, as mentioned above, the expression of many of the genes encoding components of the DNA response and DNA repair machinery, such as RAD50, RAD51, BRCA1, BRCA2 and DNA-PKc are activated by MYC [151], and depletion of MYC has been shown to attenuate ATM signaling in response to DNA damage [152]. It remains to be investigated whether CDK2 and Ser-62 phosphorylation is required for these MYC activities. Clearly, more studies are warranted to delineate the role of CDK2 and MYC in DNA damage repair in normal and cancerous cells, and how the status of MYC affects this regulation. 

## 7. The CDK2/p27/SKP2 Triangle 

**Figure 3:**
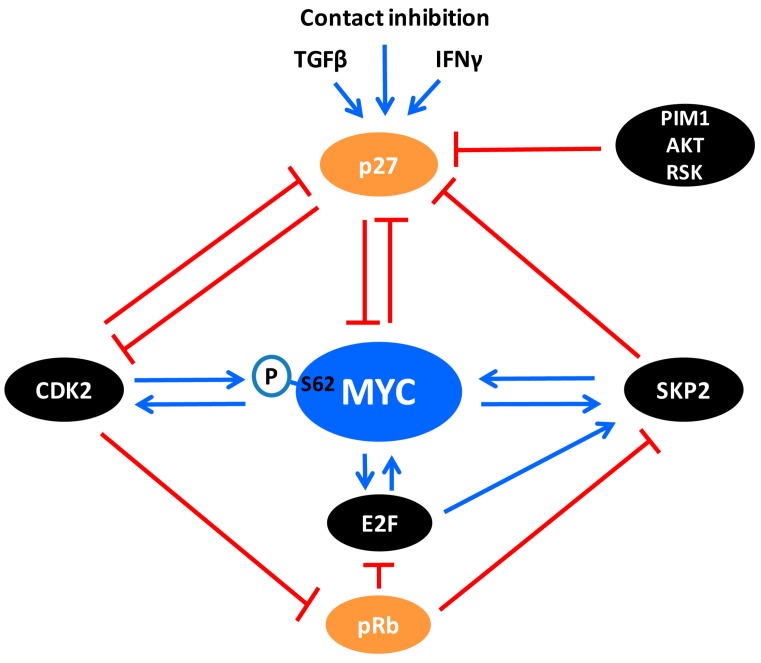
The interconnections within the MYC/CDK2/p27/SKP2 network. The positive (arrow) and negative (block sign) interconnections within the MYC/CDK2/p27/SKP2 axis are illustrated. Some additional players connected to this network, including tumor growth factor β (TGFβ), interferon-γ (IFN-γ), the kinases PIM1, AKT and RSK, and the transcription factor E2F and pRB are also depicted. The arrows can symbolize physical interactions, phosphorylation, ubiquitylation or other actions. Proteins depicted in black represent factors with growth-promoting/oncogenic functions (with the exception of MYC, which is colored blue), while those in orange represent growth/tumor-suppressive functions in this context. The growth-promoting proteins within the network are in general stimulating each other’s expression and/or activity, thus generating a positive feedback loop. In contrast, the growth-promoting and growth-suppressive proteins are in general inhibiting each other’s expression and/or activity. See the text for further explanation.

One of the gatekeepers for G1-S transition of the cell cycle is p27, which by binding to cyclin A/E-CDK2 in the nucleus blocks the catalytic activity of these complexes [153,154,155,156,157] (Figure 2 and Figure 3), thereby blocking CDK2-mediated phosphorylation of the substrates discussed in the previous section. The expression of p27 is induced by growth inhibitory signals such as tumor growth factor β (TGFβ), interferon-γ (IFN-γ), contact inhibition and loss of adhesion to extracellular matrix, as well as differentiation signals such as retinoic acid and vitamin D3 [10,158,159,160]. On the other hand, cyclin E/CDK2 can inactivate nuclear p27 by site-specific phosphorylation at threonine 187 (Thr-187), after which nuclear p27 is primed for proteasomal degradation by the SCF^SKP2^ ubiquitin ligase complex [10,122,123,124,158,159,160,161,162] (Figure 3). 

SKP2 was initially discovered as an interacting partner of the cyclin A/CDK2 complex [163], but was later identified as the substrate-binding F-box component of the multi-subunit SCF (Skp1–Cullin 1–F-box) E3 ubiquitin ligase complex SCF^SKP2^ [164,165,166]. As part of SCF^SKP2^, SKP2 binds to and targets numerous substrates, typically tumor suppressor proteins and oncoproteins linked to gene transcription, metabolism, cell survival, and cell cycle progression including, in addition to p27, p21^CIP1^, cyclin A, MYC, E2F1, p130, FOXO1, SMAD4, BRCA2, MLL, LKB1 and CDK9 [167]. The mode of ubiquitylation by SCF^SKP2^ may differ depending on target. As examples, SKP2 targets tumor suppressors such as p27 [161,162] and FOXO1 [168] through proteolytic K48-linked ubiquitylation, while LKB1 and the mTORC1-binding protein RAGA are targeted through non-proteolytic K63-linked ubiquitylation [169,170]. SKP2 also interacts with and triggers K63-linked ubiquitylation of the NBS1 subunit of the DNA damage-sensing MRE11/RAD50/NBS1 (MRN) complex upon DNA double strand breaks, leading to recruitment of ATM to the site of damage and stimulation of DNA repair by homologous recombination [171]. 

Genetic analysis using multiple *SKP2* knockout systems suggests that *SKP2* primarily functions as an oncogene [167]. For instance, *SKP2* deletion was shown to abrogate tumor formation in the pituitary and prostate in mice devoid of the major tumor suppressors p53 and pRB [172]. Numerous cancers display poor prognosis if harboring overexpressed SKP2 protein [167]. p27 has been suggested to be the most essential target of SKP2, and SKP2 and p27 are very closely linked genetically. Genetic depletion of p27 was reported to completely restore the phenotypic defects in SKP2 knockout mice [173,174]. Nakayama’s laboratory found that p27 accumulates upon SKP2 ablation in the mouse, leading to nuclear enlargement, centrosome over duplication and delayed mitosis entry, a phenomenon not occurring in SKP2/p27 double knockout mice [173,175]. The effect on mitosis suggests that p27 inhibits mitotic CDK1 in addition to CDK2 (Figure 2). Indeed, authors found that p27 associated with both CDK1 and CDK2 in SKP2 knockout MEFs, resulting in reduced cyclin-CDK activity [173]. The ability of p27 to target CDK1 was confirmed in vivo by investigating the phenotypes of double knockout CDK2/p27 mice, where depletion of CDK2 did not revert the phenotype of p27 knockout mice [176]. Expression of SKP2 and p27 are inversely correlated in many human tumors [167,177,178,179], and p27 expression also correlates inversely with cyclin-dependent kinase subunit 1 (CKS1) [18,180], a co-factor for SCF^SKP2^-induced p27 degradation [181,182]. In addition to the CUL1-containing SCF^SKP2^ complex, SKP2 can also target p27 for ubiquitylation and degradation by another SCF complex containing CUL4A and the damaged DNA-binding protein DDB1, which is associated with the COP9 signalosome, a complex with isopeptidase activity [183].

Expression and activity of SKP2 are regulated at multiple levels including mRNA expression, protein degradation and intracellular localization. SKP2 mRNA expression is activated by both E2F1 and MYC, and is under negative regulation by pRB through repression of E2F (Figure 3) [109,184,185]. Kaelin’s laboratory reported that SKP2 is degraded by the E3 ligase Anaphase-Promoting Complex (APC) containing CDH1, the activator of APC during G1 phase of the cell cycle [186]. Following up this finding, Dyson’s laboratory later demonstrated that the degradation of SKP2 by APC-CDH1 was essential for pRB-induced G1-arrest [187]. The authors showed that pRB in fact interacts with CDH1 as part of a pRB–APC–CDH1–SKP2 complex promoting SKP2 degradation, and knockdown of CDH1 abrogated pRB-induced cell cycle arrest. In addition, pRB has been reported to interact with the N-terminus of SKP2, thereby blocking the ubiquitylation of p27 as part of pRB-induced G1-arrest, occurring even prior to repression of E2F target genes by pRB [188].

## 8. The Crosstalk between MYC and SKP2

**Figure 4:**
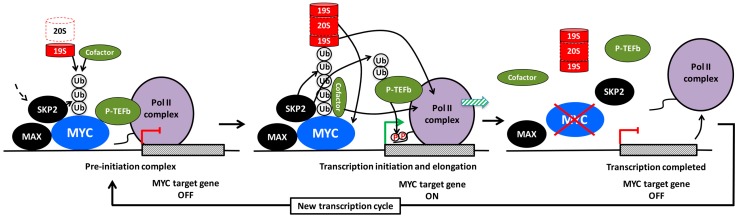
Role of ubiquitylation in MYC-driven transcription. Hypothetical model describing SKP2-mediated ubiquitylation and degradation of MYC at a MYC target gene promoter. **Left**: MYC (in complex with MAX) binds the promoter. SKP2 as part of an Skp1–Cullin1–F-box (SCF) E3 ligase complex is recruited to the promoter and starts ubiquitylating MYC. The 19S proteasome subcomplex, with or without the rest of the proteasome, and hypothetical ubiquitin-binding cofactors interacts with MYC via attraction to the growing ubiquitin chain. MYC also interacts with subunits of the RNA polymerase II (Pol II) complex and with positive transcription elongation factor P-TEFb; **Middle**: the recruited 19S complex and coactivators stimulate initiation and/or elongation of transcription. SKP2 may, as described for other transcription contexts, ubiquitylate P-TEFb and thereby contribute to transcription elongation. The ubiquitylation of MYC continues, producing longer ubiquitin chains and the 26S proteasome starts acting on MYC; **Right**: the proteasome degrades MYC and transcription is completed. SKP2, cofactors, P-TEFb and the proteasome dissociates and Pol II exits the promoter after the transcription termination site. A new round of transcription can initiate as newly synthesized MYC enters the promoter. See the text for further explanation.

As mentioned above, MYC is a direct activator of *SKP2* gene expression around the G1-S transition [109]. Further, SKP2 stabilizes the MYC protein level indirectly by promoting ubiquitylation and degradation of the E3 ligase TRUSS [189] (which targets MYC for degradation [40,44]). On the other hand, our and Tansey’s laboratory showed that the SKP2 E3 ligase complex also targets MYC for ubiquitylation and degradation during the G1/S transition of the cell cycle [190,191]. Since both MYC and SKP2 are oncoproteins of importance for S-phase entry, this finding seemed counterintuitive. Interestingly, SCF^SKP2^ and the 26S proteasome associated together with MYC at target gene promoters, indicating that ubiquitylation and degradation of MYC occurred directly at the site of MYC activity. In fact, ubiquitylation of MYC through the SCF^SKP2^ complex appeared to have dual outcomes; in addition to proteasome degradation, SKP2 also promoted and was even required for MYC-driven transcription of cell cycle-associated genes and for MYC-induced S-phase entry. This suggested that SKP2 acts as a transcriptional cofactor for MYC and feeds back to MYC in a positive forward loop where MYC and SKP2 activate each other (Figure 3 and Figure 4) [190,191]. Subsequent studies showed that SKP2 participates in the regulation of subsets of MYC target genes of importance for proliferation and cancer development [20,192,193,194,195].

As discussed earlier, both MYC depletion, CDK2 inhibition/depletion and p27 activation cause senescence induction even in p53-independent MYC-driven systems through downregulation of MYC target genes involved in senescence suppression [58,134,196]. Interestingly, Pandolfi’s laboratory reported that genetic targeting of SKP2 or pharmacological inhibition of the SCF^SKP2^ complex also induced p53-independent cellular senescence [197]. A potential role for SKP2 in MYC-dependent suppression of senescence is, however, yet to be demonstrated. 

The interaction between MYC and SKP2 is also subjected to competition from other proteins, affecting MYC’s transcriptional output. Hann’s laboratory reported that the tumor suppressor protein p19^ARF^, which is induced by MYC as a result of oncogenic stress, inhibits the interaction between MYC and SKP2 resulting in decreased MYC ubiquitylation, which in turn switches the repertoire of genes regulated by MYC towards induction of apoptosis [198]. SNIP1 is another protein that competes with SKP2 to bind MYC, thereby stabilizing MYC in the early G1 phase of the cell cycle, and possibly switching it into another mode of transcription activation through recruitment of p300 [193,199].

## 9. The Role of Ubiquitylation for MYC-Driven Transcription

The apparent connection between the transcriptional activity of MYC and its destruction by SKP2 is a good example of what has been called the “activation by destruction”, “Kamikaze” or “Black Widow” models of transcription [200,201,202,203,204]. According to this concept, destruction of the transcription factor is an integrated part of its activity, presumably as a way of keeping “dangerous” proteins under tight and timely control (Figure 4). In addition to MYC, this mechanism applies to many other oncogenic transcription factors and their cofactors, such as the estrogen, androgen, progesterone and retinoic receptors and cofactors associated with these receptors, SREBP, p53, HIF-1α and E2F-1 and the yeast transcription factors GCN4, GAL4, SPT23 and MGA2 [202,204,205]. However, ubiquitylation can also have non-proteolytic functions in regulation of transcription. Proteasomal degradation usually involves recognition of lysine 48 (K48)-linked poly-ubiquitin chains, while chains containing K63- or mixed linkages usually do not support degradation of substrates [206]. As mentioned above, SCF^SKP2^ conjugates K63-linked ubiquitin chains to certain substrates [169,170,171], while the linkage is assumed to be ubiquitin K48 in the case of MYC, although this has not been studied in detail [190,191]. Two other E3 ligases, HUWE1 (HECTH9) and FBXO28, which like SKP2 enhance MYC-driven transcription, promote ubiquitylation of MYC in a non-proteolytic manner [8,9], at least in the former case by forming K63-linked ubiquitin chains. However, HUWE1 is capable of inducing degradation of MYCN through K48-linkages [207]. The latter may reflect a substrate difference between MYC and MYCN or may depend on the cellular context, for instance what E2 subunit is engaged in different cell types.

What could be the mechanism(s) by which SKP2 and other E3 ligases promote MYC-driven transcription? There are several findings, both for MYC and other transcription factors, that gives some insight to how this might work: 

(1) Recruitment of cofactors. Ubiquitin conjugated to transcription factors in the form of mono-, oligo- or poly-ubiquitylation with various types of linkage may serve as binding sites for cofactors with a role in transcription (Figure 4, left and middle part). For example, HUWE1/HECTH9 or FBXO28-mediated poly-ubiquitylation of MYC in the MB4 region results in recruitment of the cofactor/HAT p300, which contributes to the transcription process by acetylating histones, MYC or other substrates [10,13]. Using a MYC mutant with all lysines replaced by arginines, Jaenicke et al. [20] recently showed that this mutant, although capable of binding to chromatin, was unable to recruit certain cofactors, such as TRRAP, BRD4, and P-TEFb and did not support transcription. Reintroducing a single lysine at position 52 (K52), which became ubiquitylated but not acetylated, restored recruitment of BRD4. It remains to be investigated how the conjugated ubiquitin moieties contributes mechanistically to recruitment of cofactors in this case. However, a study of the viral transcription factor VP16 showed that conjugated ubiquitin within the VP16 TAD was directly involved in the binding of P-TEFb to the TAD, thereby stimulating transcription elongation [208]. In addition to ubiquitylation of MYC, it is conceivable that recruitment of SKP2 or other E3 ligases enables these to target also other chromatin-bound factors (Figure 4, middle part). For instance, SKP2 has been reported to ubiquitylate the CDK9 subunit of P-TEFb, thereby stimulating human immunodeficiency virus (HIV) Tat-mediated transcription elongation by facilitating ternary complex formation between P-TEFb, Tat and the Tat RNA-binding site (Tar) [209].

(2) Recruitment of the proteasome. Conjugated ubiquitin chains are bound by the 26S proteasome through interaction with ubiquitin-binding subunits of the 19S regulatory subcomplex [206]. The 19S regulatory particle contains a “base” and a “lid” structure and is connected to the 20S barrel-shaped catalytic core particle. The major binding activity to ubiquitylated proteins is contributed by subunits of the “lid”. The “base” consists of a hexameric ring of AAA ATPases, the function of which is to unfold the substrate, which is subsequently fed into the hollow interior of the 20S barrel structure where it is degraded [206]. 

So, why would recruitment of the proteasome to an ubiquitylated transcription factor stimulate transcription? There are different models for how this might work: 

(a) Non-proteolytic role of the 19S regulatory subcomplex of the proteasome. There are a number of reports suggesting that the ATPase activity of the 19S AAA ATPase subunits could also participate in remodeling of transcription complexes in a non-proteolytic fashion in order to facilitate transcription (Figure 4, left and middle part). This was originally observed in yeast, where these AAA ATPases were found to be required for efficient transcription elongation, and physically interacted with elongation factors independent of the 20S core particle [210,211], and/or were involved in regulating nucleosome density in an ATPase dependent but non-proteolytic manner [212,213]. The ATP-ase activity of the 19S particle has also been shown to facilitate interactions between the SAGA HAT and Mediator complexes with promoter-bound transcription activators in yeast, thereby contributing to preinitiation complex (PIC) assembly at transcription start sites [214,215]. The AAA ATPase subunit SUG1 (RPT6) has been shown to interact with MYC and to be required for MYC-driven transcription in mammalian cells [203], but it is unclear if this occurred independently of or as part of the degradation process.

(b) Proteolytic role of the 26S proteasome. It is still not entirely clear if the 19S particle ever exist separated from the 20S proteasome particle in cells, and if it can be uncoupled from the catalytic activity of the proteasome. Studies of the distribution of 19S and 20S over the genome in yeast by ChIP suggest a very similar distribution [204]. There are numerous reports supporting the notion that the degradation is a necessary step in the transcription process—at the initiation, elongation and/or termination step—which may be necessary in order to restart another transcription cycle and thereby enable continued cycles of transcription (Figure 4, middle and right part). Both ubiquitylation and catalytic proteasome function seem to be required for MYC-driven transcription [20,216]. Further, cyclic turnover of the estrogen receptor α (ERα) receptor by the proteasome was demonstrated to be required both at ERα-induced initiation and elongation of transcription [217,218]. Also ubiquitin-dependent exchange of corepressor and coactivator complexes through proteasome-mediated degradation as part of transcription activation was reported for LIM homeodomain proteins and nuclear receptors [219,220]. According to a model proposed by Wu et al. [221] for the coactivator SRC-3, a phospho-dependent ubiquitin time clock is in operation; the interacting E3 ligase is sequentially building up mono-, oligo- and finally poly-ubiquitin chains on the substrate during which non-proteolytic transcription-promoting activities occur before the 26S proteasome terminates the process (Figure 4, middle part). It has also been suggested in the literature that different functions (ubiquitylation, ATPase activity, degradation) of the ubiquitin/proteasome system are active in different steps of the transcription cycle [222]. 

What discrete steps of the MYC-driven transcription cycle involve SKP2 and other parts of the ubiquitin/proteasome system? Although this has not been investigated specifically for SKP2, mutation of all potential ubiquitylation sites in MYC did not alter MYC’s chromatin association or RNA Pol II recruitment, but did affect recruitment of TRRAP/HATs and factors involved in transcription elongation such as BRD4 and P-TEFb [20]. These characteristics go well together with MYC’s well-established function in promoting histone acetylation and transcription elongation at promoters [7,17,19,96,223]. Further, both MYC and SKP2 have been implicated in the regulation of H3K4 trimethylation and H3K27 trimethylation and acetylation [14,15,224,225,226]—histone marks that are closely connected with actively transcribed genes and repressed genes, respectively [16]. Other described activities of SKP2 that may be relevant for MYC function, is its induction of degradation of macroH2A1, a histone variant that is associated with heterochromatin, resulting in activation of repressed genes, such as *CDK8* [227]. MacroH2A1 is together with heterochromatin protein 1 (HP1) and histone H3 lysine 9 trimethylation (H3K9me3) part of senescence-associated heterochromatin foci [228], raising the question whether this activity could be part of MYC or SKP2-mediated repression of senescence [58,196,197].

## 10. The Crosstalk between MYC and p27

**Figure 5:**
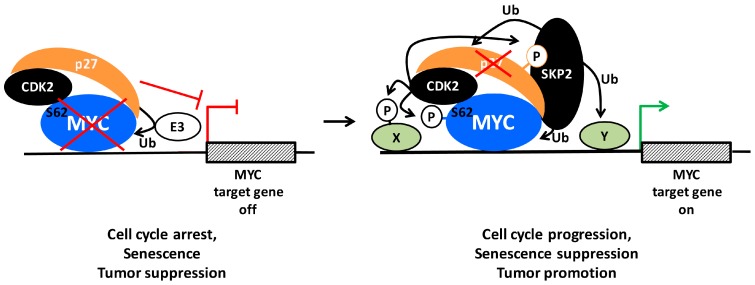
Modulation of MYC-driven transcription by the CDK2/p27/SKP2 network. Hypothetical model describing the regulation of MYC and the interplay between CDK2, p27 and SKP2 at MYC target gene promoter. **Left**: MYC (in complex with MAX) is bound at a target gene promoter and is associated with cyclin E/A/CDK2. p27 binds CDK2 and MYC and represses CDK2 activity, thereby prohibiting CDK2-mediated phosphorylation of Ser-62. p27 promotes ubiquitylation and degradation of MYC via an unidentified E3 ubiquitin ligase. p27 may possibly also actively participate in repression of transcription, for instance through association with repressor complexes as has been described in other contexts; **Right**: CDK2 activity increases during G1-S phase transition or due to oncogenic events. This leads to phosphorylation of p27, of MYC Ser-62 and possibly other substrates at the promoter. Ser-62 phosphorylation results in increased MYC activity. SKP2 recognizes phosphorylated p27 and targets it for ubiquitylation and proteasomal degradation, thereby relieving p27 repression of MYC. SKP2 also ubiquitylates MYC and possibly other substrates at the promoter, and thereby stimulates transcription (see Figure 4). The combined effects of phosphorylation and ubiquitylation of MYC and the release of p27 contributes to transcription activation. Proteins depicted in black represent factors with growth-promoting/oncogenic functions (with the exception of MYC, which is colored blue), while orange represents growth/tumor-suppressive functions in this context. See the text for further explanation.

Recent studies have revealed an interesting antagonistic crosstalk also between MYC and p27. MYC represses p27 mRNA expression by blocking FOXO3a-mediated p27 transcription [229], but also stimulates p27 protein turnover in a number of ways. As mentioned above, MYC activates transcription of SKP2, CKS1 and other components of the SCF^SKP2^ E3 ligase complex and simultaneously stimulates the activity of cyclin E/CDK2, thereby targeting p27 for proteasome-mediated degradation (Figure 2 and Figure 3). On the other hand, p27 antagonizes MYC in at least two ways (Figure 5): (1) By inhibiting CDK2 and CDK1 activity, p27 blocks phosphorylation of MYC Ser-62, which is important for MYC activity and stability as discussed in previous sections; (2) Our laboratory recently demonstrated that p27 binds to and inhibits MYC also independently of CDK2, involving a region of MYC overlapping with MB4 and engaging the C-terminus of p27 [125]. This part of p27 does not participate in binding to or inhibiting CDK2, but contains multiple phosphorylation sites, including Thr-187 targeted by CDK2 and different sites phosphorylated by the kinases PIM1, RSK and AKT—the latter ones are important for nuclear export of p27 (Figure 3) [10,158,159,230,231,232]. However, the interaction between MYC and p27 was shown to take place in the nucleus and localized to chromatin [125]. 

How does the interaction with p27 affect MYC? IFN-γ-induced association of p27 with MYC at chromatin correlated with loss of total and Ser-62-phosphorylated MYC from target promoters, reduced expression of MYC target genes, growth arrest, differentiation and induction of senescence in myeloid tumor cells [58,125]. Ectopic or signal-induced expression of p27 was also followed by reduced MYC protein levels through increased turnover via the ubiquitin/proteasome system. Intriguingly, this was independent of the cyclin/CDK-binding domain of p27 but required the C-terminal domain, suggesting that this effect of p27 is linked to its binding to MYC and distinct from its canonical cell-cycle inhibitory function. This view is further supported by the observation that p27-induced senescence of MYC + RAS-transformed rodent fibroblasts was independent of Ser-62 phosphorylation status, likely as a result of induced MYC degradation [58,125]. 

Further, since p27-induced degradation of MYC seemed to be independent of the SKP2 and FBXW7 E3 ligase complexes, these results points to the existence of a-yet-to-be-identified proteolytic E3 ligase whose activity towards MYC is mediated through p27 (Figure 5, left part). Another possibility that cannot be excluded is that p27 binding displaces stabilizing proteins associating with MYC. The interaction with p27 occurs in a part of MYC overlapping with MB4 and the basic DNA-binding region, which contains multiple ubiquitylation/acetylation and phosphorylation sites [8,9,11,233,234,235,236]. Two E3 ligases, HUWE1/HECTH9 and FBXO28, stimulates non-proteolytic ubiquitylation in this region, resulting in recruitment of p300, as mentioned above [8,9]. Binding of FBXO28 to this region is dependent on phosphorylation of FBXO28 by CDK2, suggesting that binding of p27 and FBXO28 occur during different phases of the cell cycle. Given that E3 ligases can act either through proteolytic or non-proteolytic ubiquitylation [206], as described for SKP2 above, it is conceivable that E3s previously described as non-proteolytic towards MYC, such as HUWE1/HECTH9, could have a proteolytic role in a distinct context. Interestingly, HUWE1/HECTH9 was reported to target MYCN for proteolytic degradation [207], and might have this ability against MYC as well. In addition, the SIRT1 protein deacetylase, which also binds MYC overlapping the MB4 region, deacetylates lysine residues in this region, thereby promoting non-proteolytic ubiquitylation, stabilization and enhanced MYC activity [11]. SIRT1 has been reported as a negative regulator of p27 [237], and silencing of SIRT1 induced cellular senescence in a MYC-dependent manner [11], suggesting a possible competition between SIRT1 and p27 in binding to the MB4 region of MYC. In addition, the MB4 region is phosphorylated by PIM and CK2 kinases [62,238], in the former case resulting in MYC stabilization. PIM and CK2 also phosphorylate p27, resulting in nuclear export and degradation, respectively [10,158,159,232,239]. Clearly, future studies are warranted to resolve the conundrum of how MYC protein stability is regulated by p27. 

An equally interesting possibility is that p27 more directly regulates the transcriptional activity of MYC (also in a CDK2/Ser-62-independent manner). As discussed above, binding of p27 to MYC may possibly compete with the binding of FBXO28, HUWE1/HECTH9 and SIRT1, which all stimulate MYC-induced transactivation, in the former cases by enhancing recruitment of p300. In this way, the outcome of p27 binding to MYC would be two-fold; reduced transcription of MYC target genes and degradation of MYC (Figure 5, left part). The notion of a more direct role of p27 in regulating transcription is further supported by recent publications suggesting that p27 interacts with the E2F4/p130 complex through its C-terminal domain and contributes to transcription repression by recruitment of SIN3/HDAC corepressor complexes [240]. It is conceivable that such a repression/degradation mechanism is similar (but opposite) to the “activation by destruction” or “Kamikaze” model discussed for SKP2 above, namely that binding of p27 to MYC would create a time window where transcription repressor proteins are recruited by p27 to target promoters prior to destruction of MYC and subsequent loss of p27 from the promoter (Figure 4 and Figure 5). One should however point out that there is no evidence at present that p27-induced turnover of MYC takes place at promoters. In any case, degradation of MYC may facilitate replacement of MYC with members of the MXD(MAD)-family of transcriptional repressors, which competes with MYC for binding MAX and recruits HDAC-containing co-repressor complexes to chromatin [3,5,42]. In fact, this could be one of the reasons why p27 and MXD1 cooperate in promoting terminal granulocytic differentiation, which is linked to down-regulation of MYC and cyclin E/CDK2 activity [241]. The crosstalk between MYC and p27 is also supported in vivo in p27 knockout mice, where lymphoma development was shown to co-occur with activation of MYC while such synergy could not be observed in p27 WT mice [242], thus strengthening the notion that p27 provides a negative feedback to MYC. 

Considering that p27 is a target for CDK2 and SKP2, and that all three proteins can associate with MYC at chromatin, an intriguing possibility is that p27-mediated repression of MYC at target gene promoters is reversed during G1-S phase transition or in tumors through elevated cyclin E and/or SKP2 expression. In such a scenario, cyclin E/CDK2-induced phosphorylation of p27 would be followed by SKP2-mediated degradation and loss of p27 from promoters. Through phosphorylation of Ser-62 and ubiquitylation of MYC and possibly other targets at promoters, cyclin E/CDK2 and SKP2 would subsequently contribute to further activation of transcription (Figure 5, right part). The opposite scenario would take place in response to growth inhibitory signals such as IFN-γ, which induce high levels of p27, leading to inhibition of CDK2, p27 binding to MYC, MYC degradation and reversal to a repressed state of transcription, cell cycle exit, differentiation and/or senescence. 

It should be emphasized that this model is hypothetical; it remains to be demonstrated whether p27, CDK2 and SKP2 coexist at the same promoters simultaneously. Future studies need to dissect how these factors are distributed across the genome, if they are co-dependent and what influence they have on the transcriptome in different cellular contexts, including cell cycle and senescence regulation. 

## 11. Targeting the MYC/CDK2/SKP2/p27 Axis in Cancer

Does the crosstalk between MYC and p27 described above have any relevance for human cancer? Since p27 expression is predominantly regulated at the level of mRNA translation and protein turnover [158] and p27 down-regulates MYC protein (but not mRNA) level, we investigated expression of MYC and p27 protein levels in publicly available proteomics breast cancer data from The Cancer Genome Atlas (TCGA). We found a significant inverse correlation between MYC and p27 levels in breast cancer, which was particularly strong if looking at tumors with high p27 levels concomitant with low levels of phosphorylation at Threonine 157 (Thr-157) [125]. Thr-157 is targeted by the AKT, RSK and PIM1 kinases and redirects p27 to the cytoplasm (Figure 3) [10,158,159,230,231,232]. Low levels of phosphorylation at this site are therefore an indication of a higher fraction of nuclear p27 and hence an expected lower level of MYC protein as a result of increased MYC turnover. This correlation therefore is consistent with our finding that nuclear p27 interacts with MYC and targets it for degradation. Further, high p27 levels correlated with low levels of phosphorylated pRB, which is an indication of low CDK2 activity [125]. These observations are consistent with a recent report looking at MYC, p27 and phospho-Rb expression in breast and ovarian cancers [243]. Inverse correlations between MYC and p27 protein levels have also been observed in other disease models including chronic lymphocytic leukemia and gastritis [244,245]. 

While MYC is a bona fide oncogene transcribed from the most frequently amplified locus across human cancer types, p27 does not exhibit the typical tumor suppressor properties, mostly obvious from its chromosomal locus not displaying focal and frequent deletions in cancers [34]. This is plausibly explained by the opposite roles of nuclear and cytoplasmic p27 in human cancers. While nuclear p27 acts as a break on the cell cycle by inhibiting CDK2 [101] and MYC [125] as discussed here, cytoplasmic p27 promotes cancer cell migration and invasion [246,247]. Thus, when looking at the pool of p27 harboring tumor suppressive properties it is critical to examine nuclear p27 devoid of phosphorylations at C-terminal sites including Thr-157. The protein data sets in TCGA are getting constantly updated and should therefore contain sufficient material to study the relationships between MYC and nuclear p27 in the near future in a broader range of human tumors.

In addition to the relationship between MYC and p27, there is a well-established inverse correlation between SKP2 and p27 the expression in many human tumors as mentioned above, including thyroid, oral, breast, prostate, ovarian and lung cancer as well as lymphoma [10,158,159,160,167,177,178,179]. Overexpression of cyclin E is another cause of p27 downregulation, and occurs in several tumor types such as ovarian, uterine cancer, colorectal and bladder cancers as well as T-ALL, and can be caused by gene amplification or protein stabilization due to FBXW7 loss [34,72,248]. 

What possibilities are there to target the MYC/CDK2/SKP2/p27 axis in cancer, i.e., reducing the activity of MYC, CDK2 or SKP2, or boosting nuclear p27 expression? There are numerous potential ways that can be considered. One is to utilize pharmacological inhibitors of CDK1/2. Treatment with CDK1/2 inhibitors or depletion of CDK2 efficiently inhibit growth and/or induce apoptosis in triple negative/basal breast cancer, *MYCN*-amplified neuroblastoma and myeloid leukemia cell lines [58,134,249,250] and in mouse models of MYC-driven lymphoma and liver cancer [134,249]. Clinical trials using various CDK1/2-inhibitors have produced some encouraging results in hematopoietic malignancies, but have not been very effective in solid tumors compared with the more successful clinical outcomes from targeting their siblings CDK4/6 [248]. While this may be due to non-optimal patient stratification or limited tumor uptake, CDK2-inhibitors generally suffer from specificity problems. In fact, CDK2 inhibitors available in the clinic today are non-selective and also target CDK1 and CDK9 among other CDKs, causing side effects that reduce the therapeutic window of these drugs. Improved, more selective CDK2 inhibitors are therefore warranted. The MYC/CDK2/SKP2/p27 axis could also be therapeutically exploited on the RNA-level. One of us demonstrated recently that in vivo administration of microRNAs evolutionarily enriched to target multiple cyclins/CDKs, including cyclin E and CDK2, efficiently blunts the progression of patient-derived, treatment-refractory triple negative/basal breast cancers without detectable animal toxicity [251,252]. Another potential strategy is inhibition of cellular PP2A inhibitors using SET antagonist peptides, thereby reducing Ser-62 phosphorylation and MYC stability [253]. Other interesting approaches are to utilize small molecule inhibitors of the p27/SKP2 interaction, which stabilizes p27 [254,255,256]. Inhibitors blocking AKT, PIM1 and RSK kinases phosphorylating the C-terminus of p27 [10,158,159,230,231,232], which would favor nuclear accumulation of p27, are under clinical development [61,257,258]. With respect to targeting of MYC, downregulation of MYC mRNA expression can be achieved by bromodomain and extra-terminal motif (BET) inhibitors in certain tumor types, while targeting the PI3K/mTOR pathway is known to affect both MYC turnover and mRNA translation [46,47,75,259]. Attempts to target the MYC protein directly, for instance by small molecules interfering with the interaction between MYC and MAX, have been challenging and have not reached the clinic, but may be an option in the future [259,260,261].

Finally, the induction of p27 by cytokines like IFN-γ and TGF-β or by differentiation-, cell density- and adhesion-signals [10,158,159] has been shown to inhibit growth of MYC-driven tumor cells in different systems [58,241,262]. IFN-γ has been used in a broad range of clinical trials since the late 1980s [263] but its clinical use is, however, being limited due to adverse effects when over-stimulating the immune system [264]. IFN-γ is produced by CD4+ Th1 T-lymphocytes and plays an important role in immunosurveillance of tumors by activating cytotoxic T-cells that eliminates tumor cells [265]. However, IFN-γ and other cytokines produced by CD4+ Th1 T-cell can also target tumor cells directly thereby keeping tumors in check [266], possibly by inducing p27 as discussed here. Interestingly, CD4+ T-cells play a crucial role in eliminating tumors after MYC depletion in a Tet-driven MYC on/off mouse model system [267]. Immunotherapy has reached new levels thanks to cutting edge approaches to boost the innate and adaptive immune system, for instance through checkpoint blockade and vaccine development [268]. Technological advances in methods for purification, expansion and universal or tumor-specific activation of patient-derived T-cells could pave the way towards the elimination of MYC-driven tumors through local production of IFN-γ by CD4+ T-helper cells, which potentially would have the dual effect of upregulation of p27 expression in tumor cells together with increased immunosurveillance. Irvine’s laboratory used an ex vivo approach of expanding primary T-cells, able to differentiate into both CD4+ and CD8+ T-cells, subsequently followed by surface coating of these cells by nanoparticles containing the chemotherapeutic drug SN-38. Administration of such nanoparticle-loaded T-cells to mice with aggressive MYC-driven lymphoma resulted in several magnitudes of improved drug uptake in tumor-bearing lymph nodes leading to tumor regression [269]. Utilizing T-cell therapy combined with nanoparticles carrying next-generation drugs or RNAi targeting components of the MYC/CDK2/SKP2/p27 axis may be a new future solution to combat MYC-driven tumors.

## 12. Concluding Remarks and Future Perspectives

One of the most prominent oncogenic features of MYC is its ability to promote cell proliferation despite unfavorable conditions, which causes severe cellular stress, usually leading to cell death. This clearly limits MYC’s oncogenic potential. MYC therefore needs to cooperate with other factors in order to overcome these limitations, such as oncogenic RAS, the anti-apoptotic members of the BCL-2 family and mutated p53. This review has focused on the role of the CDK2/p27/SKP2 network, which is regulated by MYC but also feeds back to MYC through protein–protein interactions and post-translational modifications in positive or negative loops, thereby modulating MYC’s ability to regulate genes involved in cell cycle progression and senescence suppression.

There are a number of questions that remains to be answered concerning the interplay between MYC and the CDK2/p27/SKP2 network. It is still unclear what subsets of MYC target genes are under control of this network. Considering the well-documented role of CDK2, p27 and SKP2 in cell cycle regulation, genes involved in this process are good candidates, and examples of such genes have also been documented [58,125,191]. However, maintaining the cell cycle and suppression of senescence is not only about stimulating G1-S transition and other phases of the cell cycle, but also about coping with oncogene-induced stress. As discussed in this review, there are several indications that MYC Ser-62 phosphorylation and functions of CDK2, SKP2 and p27 are connected to coping with replicative and oxidative stress. One option is therefore that the role of the positive and negative regulators within this network is to maintain MYC-driven cell cycle progression under controlled and “safe” conditions. Further characterization of subsets of MYC target genes and cellular processes controlled by the MYC/CDK2/p27/SKP2 axis could be achieved, for instance, by genome-wide ChIP and RNA-sequencing and proteomics. Other issues for future research are how Ser-62 phosphorylation impacts on MYC activity mechanistically, how p27 promotes MYC degradation and whether p27 also plays a transcriptional role. It also remains to be seen whether SKP2 targets p27 in the context of a ternary complex with MYC, whether SKP2-induced ubiquitylation and degradation of p27 and MYC occurs at chromatin as part of a coactivator function and how this affects MYC-driven transcription mechanistically. Finally, many challenges lie ahead on how to efficiently and specifically target the components of the MYC/CDK2/p27/SKP2 axis for improved cancer treatment. 

## References

[1] Blackwood E.M., Eisenman R.N. (1991). Max: A helix-loop-helix zipper protein that forms a sequence-specific DNA-binding complex with myc. Science.

[2] Dang C.V. (2012). MYC on the path to cancer. Cell.

[3] Eilers M., Eisenman R.N. (2008). MYC’s broad reach. Genes Dev..

[4] Larsson L.G., Henriksson M.A. (2010). The yin and yang functions of the MYC oncoprotein in cancer development and as targets for therapy. Exp. Cell Res..

[5] Meyer N., Penn L.Z. (2008). Reflecting on 25 years with MYC. Nat. Rev. Cancer.

[6] Tu W.B., Helander S., Pilstal R., Hickman K.A., Lourenco C., Jurisica I., Raught B., Wallner B., Sunnerhagen M., Penn L.Z. (2015). MYC and its interactors take shape. Biochim. Biophys. Acta.

[7] McMahon S.B., Van Buskirk H.A., Dugan K.A., Copeland T.D., Cole M.D. (1998). The novel ATM-related protein TRRAP is an essential cofactor for the c-myc and e2f oncoproteins. Cell.

[8] Adhikary S., Marinoni F., Hock A., Hulleman E., Popov N., Beier R., Bernard S., Quarto M., Capra M., Goettig S. (2005). The ubiquitin ligase hectH9 regulates transcriptional activation by Myc and is essential for tumor cell proliferation. Cell.

[9] Cepeda D., Ng H.F., Sharifi H.R., Mahmoudi S., Cerrato V.S., Fredlund E., Magnusson K., Nilsson H., Malyukova A., Rantala J. (2013). Cdk-mediated activation of the scf(fbxo) (28) ubiquitin ligase promotes myc-driven transcription and tumourigenesis and predicts poor survival in breast cancer. EMBO Mol. Med..

[10] Vervoorts J., Luscher B. (2008). Post-translational regulation of the tumor suppressor p27(kip1). Cell. Mol. Life Sci..

[11] Menssen A., Hydbring P., Kapelle K., Vervoorts J., Diebold J., Luscher B., Larsson L.G., Hermeking H. (2012). The c-myc oncoprotein, the nampt enzyme, the sirt1-inhibitor dbc1, and the sirt1 deacetylase form a positive feedback loop. Proc. Natl. Acad. Sci. USA.

[12] Zhang K., Faiola F., Martinez E. (2005). Six lysine residues on c-myc are direct substrates for acetylation by p300. Biochem. Biophys. Res. Commun..

[13] Li B., Carey M., Workman J.L. (2007). The role of chromatin during transcription. Cell.

[14] Thomas L.R., Wang Q., Grieb B.C., Phan J., Foshage A.M., Sun Q., Olejniczak E.T., Clark T., Dey S., Lorey S. (2015). Interaction with wdr5 promotes target gene recognition and tumorigenesis by myc. Mol. Cell.

[15] Ullius A., Luscher-Firzlaff J., Costa I.G., Walsemann G., Forst A.H., Gusmao E.G., Kapelle K., Kleine H., Kremmer E., Vervoorts J. (2014). The interaction of myc with the trithorax protein ash2l promotes gene transcription by regulating h3k27 modification. Nucleic Acids Res..

[16] Shilatifard A. (2012). The compass family of histone H3K4 methylases: Mechanisms of regulation in development and disease pathogenesis. Annu. Rev. Biochem..

[17] Eberhardy S.R., Farnham P.J. (2002). Myc recruits p-tefb to mediate the final step in the transcriptional activation of the cad promoter. J. Biol. Chem..

[18] Kawakami K., Enokida H., Tachiwada T., Nishiyama K., Seki N., Nakagawa M. (2007). Increased SKP2 and CKS1 gene expression contributes to the progression of human urothelial carcinoma. J. Urol..

[19] Rahl P.B., Lin C.Y., Seila A.C., Flynn R.A., McCuine S., Burge C.B., Sharp P.A., Young R.A. (2010). C-myc regulates transcriptional pause release. Cell.

[20] Jaenicke L.A., von Eyss B., Carstensen A., Wolf E., Xu W., Greifenberg A.K., Geyer M., Eilers M., Popov N. (2016). Ubiquitin-dependent turnover of myc antagonizes myc/paf1c complex accumulation to drive transcriptional elongation. Mol. Cell.

[21] Feng X.H., Liang Y.Y., Liang M., Zhai W., Lin X. (2002). Direct interaction of c-Myc with Smad2 and Smad3 to inhibit tgf-beta-mediated induction of the cdk inhibitor p15(ink4b). Mol. Cell.

[22] Gartel A.L., Ye X., Goufman E., Shianov P., Hay N., Najmabadi F., Tyner A.L. (2001). Myc represses the p21(waf1/cip1) promoter and interacts with sp1/sp3. Proc. Natl. Acad. Sci. USA.

[23] Peukert K., Staller P., Schneider A., Carmichael G., Hanel F., Eilers M. (1997). An alternative pathway for gene regulation by myc. EMBO J..

[24] Seoane J., Pouponnot C., Staller P., Schader M., Eilers M., Massague J. (2001). Tgfbeta influences myc, miz-1 and smad to control the cdk inhibitor p15ink4b. Nat. Cell Biol..

[25] Staller P., Peukert K., Kiermaier A., Seoane J., Lukas J., Karsunky H., Moroy T., Bartek J., Massague J., Hanel F. (2001). Repression of p15ink4b expression by myc through association with miz-1. Nat. Cell Biol..

[26] Walz S., Lorenzin F., Morton J., Wiese K.E., von Eyss B., Herold S., Rycak L., Dumay-Odelot H., Karim S., Bartkuhn M. (2014). Activation and repression by oncogenic myc shape tumour-specific gene expression profiles. Nature.

[27] Wu S., Cetinkaya C., Munoz-Alonso M.J., von der Lehr N., Bahram F., Beuger V., Eilers M., Leon J., Larsson L.G. (2003). Myc represses differentiation-induced p21cip1 expression via miz-1-dependent interaction with the p21 core promoter. Oncogene.

[28] Garcia-Sanz P., Quintanilla A., Lafita M.C., Moreno-Bueno G., Garcia-Gutierrez L., Tabor V., Varela I., Shiio Y., Larsson L.G., Portillo F. (2014). Sin3b interacts with myc and decreases myc levels. J. Biol. Chem..

[29] Kurland J.F., Tansey W.P. (2008). Myc-mediated transcriptional repression by recruitment of histone deacetylase. Cancer Res..

[30] Kress T.R., Sabo A., Amati B. (2015). Myc: Connecting selective transcriptional control to global RNA production. Nat. Rev. Cancer.

[31] Lin C.Y., Loven J., Rahl P.B., Paranal R.M., Burge C.B., Bradner J.E., Lee T.I., Young R.A. (2012). Transcriptional amplification in tumor cells with elevated c-myc. Cell.

[32] Nie Z., Hu G., Wei G., Cui K., Yamane A., Resch W., Wang R., Green D.R., Tessarollo L., Casellas R. (2012). C-myc is a universal amplifier of expressed genes in lymphocytes and embryonic stem cells. Cell.

[33] Sabo A., Kress T.R., Pelizzola M., de Pretis S., Gorski M.M., Tesi A., Morelli M.J., Bora P., Doni M., Verrecchia A. (2014). Selective transcriptional regulation by myc in cellular growth control and lymphomagenesis. Nature.

[34] Beroukhim R., Mermel C.H., Porter D., Wei G., Raychaudhuri S., Donovan J., Barretina J., Boehm J.S., Dobson J., Urashima M. (2010). The landscape of somatic copy-number alteration across human cancers. Nature.

[35] Levens D. (2010). You don’t muck with Myc. Genes Cancer.

[36] Loven J., Hoke H.A., Lin C.Y., Lau A., Orlando D.A., Vakoc C.R., Bradner J.E., Lee T.I., Young R.A. (2013). Selective inhibition of tumor oncogenes by disruption of super-enhancers. Cell.

[37] Sur I.K., Hallikas O., Vaharautio A., Yan J., Turunen M., Enge M., Taipale M., Karhu A., Aaltonen L.A., Taipale J. (2012). Mice lacking a myc enhancer that includes human snp rs6983267 are resistant to intestinal tumors. Science.

[38] Wierstra I., Alves J. (2008). The c-myc promoter: Still mystery and challenge. Adv. Cancer Res..

[39] Zhang X., Choi P.S., Francis J.M., Imielinski M., Watanabe H., Cherniack A.D., Meyerson M. (2016). Identification of focally amplified lineage-specific super-enhancers in human epithelial cancers. Nat. Genet..

[40] Farrell A.S., Sears R.C. (2014). Myc Degradation. Cold Spring Harb. Perspect. Med..

[41] Kalkat M., Chan P.K., Wasylishen A.R., Srikumar T., Kim S.S., Ponzielli R., Bazett-Jones D.P., Raught B., Penn L.Z. (2014). Identification of c-myc sumoylation by mass spectrometry. PLoS ONE.

[42] Luscher B., Vervoorts J. (2012). Regulation of gene transcription by the oncoprotein myc. Gene.

[43] Sabo A., Doni M., Amati B. (2014). Sumoylation of myc-family proteins. PLoS ONE.

[44] Thomas L.R., Tansey W.P. (2011). Proteolytic control of the oncoprotein transcription factor myc. Adv. Cancer Res..

[45] Bhat M., Robichaud N., Hulea L., Sonenberg N., Pelletier J., Topisirovic I. (2015). Targeting the translation machinery in cancer. Nat. Rev. Drug Discov..

[46] Castell A., Larsson L.G. (2015). Targeting myc translation in colorectal cancer. Cancer Discov..

[47] Wiegering A., Uthe F.W., Jamieson T., Ruoss Y., Huttenrauch M., Kuspert M., Pfann C., Nixon C., Herold S., Walz S. (2015). Targeting translation initiation bypasses signaling crosstalk mechanisms that maintain high myc levels in colorectal cancer. Cancer Discov..

[48] Wolfe A.L., Singh K., Zhong Y., Drewe P., Rajasekhar V.K., Sanghvi V.R., Mavrakis K.J., Jiang M., Roderick J.E., Van der Meulen J. (2014). Rna g-quadruplexes cause eif4a-dependent oncogene translation in cancer. Nature.

[49] Hann S.R. (2006). Role of post-translational modifications in regulating c-myc proteolysis, transcriptional activity and biological function. Semin. Cancer Biol..

[50] Benassi B., Fanciulli M., Fiorentino F., Porrello A., Chiorino G., Loda M., Zupi G., Biroccio A. (2006). C-myc phosphorylation is required for cellular response to oxidative stress. Mol. Cell.

[51] Lutterbach B., Hann S.R. (1994). Hierarchical phosphorylation at N-terminal transformation-sensitive sites in c-myc protein is regulated by mitogens and in mitosis. Mol. Cell. Biol..

[52] Noguchi K., Kitanaka C., Yamana H., Kokubu A., Mochizuki T., Kuchino Y. (1999). Regulation of c-myc through phosphorylation at Ser-62 and Ser-71 by c-Jun N-terminal kinase. J. Biol. Chem..

[53] Sears R., Nuckolls F., Haura E., Taya Y., Tamai K., Nevins J.R. (2000). Multiple ras-dependent phosphorylation pathways regulate myc protein stability. Genes Dev..

[54] Seth A., Alvarez E., Gupta S., Davis R.J. (1991). A phosphorylation site located in the NH2-terminal domain of c-myc increases transactivation of gene expression. J. Biol. Chem..

[55] Watnick R.S., Rodriguez R.K., Wang S., Blois A.L., Rangarajan A., Ince T., Weinberg R.A. (2015). Thrombospondin-1 repression is mediated via distinct mechanisms in fibroblasts and epithelial cells. Oncogene.

[56] Yeh E., Cunningham M., Arnold H., Chasse D., Monteith T., Ivaldi G., Hahn W.C., Stukenberg P.T., Shenolikar S., Uchida T. (2004). A signalling pathway controlling c-Myc degradation that impacts oncogenic transformation of human cells. Nat. Cell Biol..

[57] Hoang A.T., Lutterbach B., Lewis B.C., Yano T., Chou T.Y., Barrett J.F., Raffeld M., Hann S.R., Dang C.V. (1995). A link between increased transforming activity of lymphoma-derived myc mutant alleles, their defective regulation by p107, and altered phosphorylation of the c-Myc transactivation domain. Mol. Cell. Biol..

[58] Hydbring P., Bahram F., Su Y., Tronnersjo S., Hogstrand K., von der Lehr N., Sharifi H.R., Lilischkis R., Hein N., Wu S. (2010). Phosphorylation by Cdk2 is required for Myc to repress Ras-induced senescence in cotransformation. Proc. Natl. Acad. Sci. USA.

[59] Seo H.R., Kim J., Bae S., Soh J.W., Lee Y.S. (2008). Cdk5-mediated phosphorylation of c-Myc on Ser-62 is essential in transcriptional activation of cyclin b1 by cyclin g1. J. Biol. Chem..

[60] Sjostrom S.K., Finn G., Hahn W.C., Rowitch D.H., Kenney A.M. (2005). The cdk1 complex plays a prime role in regulating n-myc phosphorylation and turnover in neural precursors. Dev. Cell.

[61] Horiuchi D., Camarda R., Zhou A.Y., Yau C., Momcilovic O., Balakrishnan S., Corella A.N., Eyob H., Kessenbrock K., Lawson D.A. (2016). Pim1 kinase inhibition as a targeted therapy against triple-negative breast tumors with elevated myc expression. Nat. Med..

[62] Zhang Y., Wang Z., Li X., Magnuson N.S. (2008). Pim kinase-dependent inhibition of c-Myc degradation. Oncogene.

[63] Gregory M.A., Qi Y., Hann S.R. (2003). Phosphorylation by glycogen synthase kinase-3 controls c-Myc proteolysis and subnuclear localization. J. Biol. Chem..

[64] Welcker M., Orian A., Jin J., Grim J.E., Harper J.W., Eisenman R.N., Clurman B.E. (2004). The fbw7 tumor suppressor regulates glycogen synthase kinase 3 phosphorylation-dependent c-Myc protein degradation. Proc. Natl. Acad. Sci. USA.

[65] Yada M., Hatakeyama S., Kamura T., Nishiyama M., Tsunematsu R., Imaki H., Ishida N., Okumura F., Nakayama K., Nakayama K.I. (2004). Phosphorylation-dependent degradation of c-Myc is mediated by the f-box protein fbw7. EMBO J..

[66] Cardozo T., Pagano M. (2004). The scf ubiquitin ligase: Insights into a molecular machine. Nat. Rev. Mol. Cell Biol..

[67] Bahram F., von der Lehr N., Cetinkaya C., Larsson L.G. (2000). C-myc hot spot mutations in lymphomas result in inefficient ubiquitination and decreased proteasome-mediated turnover. Blood.

[68] Gregory M.A., Hann S.R. (2000). C-myc proteolysis by the ubiquitin-proteasome pathway: Stabilization of c-myc in burkitt’s lymphoma cells. Mol. Cell. Biol..

[69] Hemann M.T., Bric A., Teruya-Feldstein J., Herbst A., Nilsson J.A., Cordon-Cardo C., Cleveland J.L., Tansey W.P., Lowe S.W. (2005). Evasion of the p53 tumour surveillance network by tumour-derived Myc mutants. Nature.

[70] Salghetti S.E., Kim S.Y., Tansey W.P. (1999). Destruction of myc by ubiquitin-mediated proteolysis: Cancer-associated and transforming mutations stabilize Myc. EMBO J..

[71] Akhoondi S., Sun D., von der Lehr N., Apostolidou S., Klotz K., Maljukova A., Cepeda D., Fiegl H., Dafou D., Marth C. (2007). Fbxw7/hcdc4 is a general tumor suppressor in human cancer. Cancer Res..

[72] Davis R.J., Welcker M., Clurman B.E. (2014). Tumor suppression by the Fbw7 ubiquitin ligase: Mechanisms and opportunities. Cancer Cell.

[73] Manning B.D., Toker A. (2017). Akt/pkb signaling: Navigating the network. Cell.

[74] Kenney A.M., Cole M.D., Rowitch D.H. (2003). Nmyc upregulation by sonic hedgehog signaling promotes proliferation in developing cerebellar granule neuron precursors. Development.

[75] Chesler L., Schlieve C., Goldenberg D.D., Kenney A., Kim G., McMillan A., Matthay K.K., Rowitch D., Weiss W.A. (2006). Inhibition of phosphatidylinositol 3-kinase destabilizes mycn protein and blocks malignant progression in neuroblastoma. Cancer Res..

[76] Kumar A., Marques M., Carrera A.C. (2006). Phosphoinositide 3-kinase activation in late G1 is required for c-Myc stabilization and s phase entry. Mol. Cell. Biol..

[77] Arnold H.K., Zhang X., Daniel C.J., Tibbitts D., Escamilla-Powers J., Farrell A., Tokarz S., Morgan C., Sears R.C. (2009). The axin1 scaffold protein promotes formation of a degradation complex for c-Myc. EMBO J..

[78] Zhang X., Farrell A.S., Daniel C.J., Arnold H., Scanlan C., Laraway B.J., Janghorban M., Lum L., Chen D., Troxell M. (2012). Mechanistic insight into myc stabilization in breast cancer involving aberrant axin1 expression. Proc. Natl. Acad. Sci. USA.

[79] Junttila M.R., Puustinen P., Niemela M., Ahola R., Arnold H., Bottzauw T., Ala-aho R., Nielsen C., Ivaska J., Taya Y. (2007). Cip2a inhibits pp2a in human malignancies. Cell.

[80] Khanna A., Pimanda J.E., Westermarck J. (2013). Cancerous inhibitor of protein phosphatase 2A, an emerging human oncoprotein and a potential cancer therapy target. Cancer Res..

[81] Chang D.W., Claassen G.F., Hann S.R., Cole M.D. (2000). The c-Myc transactivation domain is a direct modulator of apoptotic versus proliferative signals. Mol. Cell. Biol..

[82] Pulverer B.J., Fisher C., Vousden K., Littlewood T., Evan G., Woodgett J.R. (1994). Site-specific modulation of c-Myc cotransformation by residues phosphorylated in vivo. Oncogene.

[83] Henriksson M., Bakardjiev A., Klein G., Luscher B. (1993). Phosphorylation sites mapping in the N-terminal domain of c-Myc modulate its transforming potential. Oncogene.

[84] Wang X., Cunningham M., Zhang X., Tokarz S., Laraway B., Troxell M., Sears R.C. (2011). Phosphorylation regulates c-myc’s oncogenic activity in the mammary gland. Cancer Res..

[85] Myant K., Qiao X., Halonen T., Come C., Laine A., Janghorban M., Partanen J.I., Cassidy J., Ogg E.L., Cammareri P. (2015). Serine 62-phosphorylated myc associates with nuclear lamins and its regulation by cip2a is essential for regenerative proliferation. Cell Rep..

[86] Popov N., Herold S., Llamazares M., Schulein C., Eilers M. (2007). Fbw7 and usp28 regulate myc protein stability in response to DNA damage. Cell Cycle.

[87] Chou T.Y., Hart G.W., Dang C.V. (1995). C-myc is glycosylated at threonine 58, a known phosphorylation site and a mutational hot spot in lymphomas. J. Biol. Chem..

[88] Liu L., Eisenman R.N. (2012). Regulation of c-myc protein abundance by a protein phosphatase 2a-glycogen synthase kinase 3beta-negative feedback pathway. Genes Cancer.

[89] Seth A., Gupta S., Davis R.J. (1993). Cell cycle regulation of the c-myc transcriptional activation domain. Mol. Cell. Biol..

[90] Gupta S., Seth A., Davis R.J. (1993). Transactivation of gene expression by myc is inhibited by mutation at the phosphorylation sites thr-58 and ser-62. Proc. Natl. Acad. Sci. USA.

[91] Farrell A.S., Pelz C., Wang X., Daniel C.J., Wang Z., Su Y., Janghorban M., Zhang X., Morgan C., Impey S. (2013). Pin1 regulates the dynamics of c-myc DNA binding to facilitate target gene regulation and oncogenesis. Mol. Cell. Biol..

[92] Pineda-Lucena A., Ho C.S., Mao D.Y., Sheng Y., Laister R.C., Muhandiram R., Lu Y., Seet B.T., Katz S., Szyperski T. (2005). A structure-based model of the c-myc/bin1 protein interaction shows alternative splicing of bin1 and c-myc phosphorylation are key binding determinants. J. Mol. Biol..

[93] Elliott K., Sakamuro D., Basu A., Du W., Wunner W., Staller P., Gaubatz S., Zhang H., Prochownik E., Eilers M. (1999). Bin1 functionally interacts with myc and inhibits cell proliferation via multiple mechanisms. Oncogene.

[94] DuHadaway J.B., Sakamuro D., Ewert D.L., Prendergast G.C. (2001). Bin1 mediates apoptosis by c-myc in transformed primary cells. Cancer Res..

[95] Pavri R., Zhu B., Li G., Trojer P., Mandal S., Shilatifard A., Reinberg D. (2006). Histone h2b monoubiquitination functions cooperatively with fact to regulate elongation by RNA polymerase II. Cell.

[96] Bouchard C., Marquardt J., Bras A., Medema R.H., Eilers M. (2004). Myc-induced proliferation and transformation require akt-mediated phosphorylation of foxo proteins. EMBO J..

[97] Rahl P.B., Young R.A. (2014). Myc and transcription elongation. Cold Spring Harb. Perspect. Med..

[98] Kind J., Pagie L., Ortabozkoyun H., Boyle S., de Vries S.S., Janssen H., Amendola M., Nolen L.D., Bickmore W.A., van Steensel B. (2013). Single-cell dynamics of genome-nuclear lamina interactions. Cell.

[99] Ashton G.H., Morton J.P., Myant K., Phesse T.J., Ridgway R.A., Marsh V., Wilkins J.A., Athineos D., Muncan V., Kemp R. (2010). Focal adhesion kinase is required for intestinal regeneration and tumorigenesis downstream of wnt/c-myc signaling. Dev. Cell.

[100] Athineos D., Sansom O.J. (2010). Myc heterozygosity attenuates the phenotypes of apc deficiency in the small intestine. Oncogene.

[101] Hydbring P., Malumbres M., Sicinski P. (2016). Non-canonical functions of cell cycle cyclins and cyclin-dependent kinases. Nat. Rev. Mol. Cell Biol..

[102] Pelengaris S., Khan M., Evan G. (2002). C-myc: More than just a matter of life and death. Nat. Rev. Cancer.

[103] Perez-Roger I., Solomon D.L., Sewing A., Land H. (1997). Myc activation of cyclin e/cdk2 kinase involves induction of cyclin e gene transcription and inhibition of p27(kip1) binding to newly formed complexes. Oncogene.

[104] Prall O.W., Rogan E.M., Musgrove E.A., Watts C.K., Sutherland R.L. (1998). C-myc or cyclin d1 mimics estrogen effects on cyclin e-cdk2 activation and cell cycle reentry. Mol. Cell. Biol..

[105] Beier R., Burgin A., Kiermaier A., Fero M., Karsunky H., Saffrich R., Moroy T., Ansorge W., Roberts J., Eilers M. (2000). Induction of cyclin e-cdk2 kinase activity, e2f-dependent transcription and cell growth by myc are genetically separable events. EMBO J..

[106] Bouchard C., Thieke K., Maier A., Saffrich R., Hanley-Hyde J., Ansorge W., Reed S., Sicinski P., Bartek J., Eilers M. (1999). Direct induction of cyclin d2 by myc contributes to cell cycle progression and sequestration of p27. EMBO J..

[107] Hermeking H., Rago C., Schuhmacher M., Li Q., Barrett J.F., Obaya A.J., O’Connell B.C., Mateyak M.K., Tam W., Kohlhuber F. (2000). Identification of cdk4 as a target of c-myc. Proc. Natl. Acad. Sci. USA.

[108] Perez-Roger I., Kim S.H., Griffiths B., Sewing A., Land H. (1999). Cyclins d1 and d2 mediate myc-induced proliferation via sequestration of p27(kip1) and p21(cip1). EMBO J..

[109] Bretones G., Acosta J.C., Caraballo J.M., Ferrandiz N., Gomez-Casares M.T., Albajar M., Blanco R., Ruiz P., Hung W.C., Albero M.P. (2011). Skp2 oncogene is a direct myc target gene and myc down-regulates p27(kip1) through skp2 in human leukemia cells. J. Biol. Chem..

[110] Keller U.B., Old J.B., Dorsey F.C., Nilsson J.A., Nilsson L., MacLean K.H., Chung L., Yang C., Spruck C., Boyd K. (2007). Myc targets cks1 to provoke the suppression of p27kip1, proliferation and lymphomagenesis. EMBO J..

[111] O’Hagan R.C., Ohh M., David G., de Alboran I.M., Alt F.W., Kaelin W.G., DePinho R.A. (2000). Myc-enhanced expression of cul1 promotes ubiquitin-dependent proteolysis and cell cycle progression. Genes Dev..

[112] Chi Y., Welcker M., Hizli A.A., Posakony J.J., Aebersold R., Clurman B.E. (2008). Identification of cdk2 substrates in human cell lysates. Genome Biol..

[113] Hwang H.C., Clurman B.E. (2005). Cyclin e in normal and neoplastic cell cycles. Oncogene.

[114] Odajima J., Saini S., Jung P., Ndassa-Colday Y., Ficaro S., Geng Y., Marco E., Michowski W., Wang Y.E., DeCaprio J.A. (2016). Proteomic landscape of tissue-specific cyclin e functions in vivo. PLoS Genet..

[115] Cao L., Faha B., Dembski M., Tsai L.H., Harlow E., Dyson N. (1992). Independent binding of the retinoblastoma protein and p107 to the transcription factor e2f. Nature.

[116] Classon M., Harlow E. (2002). The retinoblastoma tumour suppressor in development and cancer. Nat. Rev. Cancer.

[117] Dyson N.J. (2016). Rb1: A prototype tumor suppressor and an enigma. Genes Dev..

[118] Geng Y., Eaton E.N., Picon M., Roberts J.M., Lundberg A.S., Gifford A., Sardet C., Weinberg R.A. (1996). Regulation of cyclin e transcription by e2fs and retinoblastoma protein. Oncogene.

[119] Knudsen E.S., Wang J.Y. (1996). Differential regulation of retinoblastoma protein function by specific cdk phosphorylation sites. J. Biol. Chem..

[120] Munro S., Carr S.M., La Thangue N.B. (2012). Diversity within the prb pathway: Is there a code of conduct?. Oncogene.

[121] Zarkowska T., Mittnacht S. (1997). Differential phosphorylation of the retinoblastoma protein by g1/s cyclin-dependent kinases. J. Biol. Chem..

[122] Montagnoli A., Fiore F., Eytan E., Carrano A.C., Draetta G.F., Hershko A., Pagano M. (1999). Ubiquitination of p27 is regulated by cdk-dependent phosphorylation and trimeric complex formation. Genes Dev..

[123] Sheaff R.J., Groudine M., Gordon M., Roberts J.M., Clurman B.E. (1997). Cyclin e-cdk2 is a regulator of p27kip1. Genes Dev..

[124] Vlach J., Hennecke S., Amati B. (1997). Phosphorylation-dependent degradation of the cyclin-dependent kinase inhibitor p27. EMBO J..

[125] Bahram F., Hydbring P., Tronnersjo S., Zakaria S.M., Frings O., Fahlen S., Nilsson H., Goodwin J., von der Lehr N., Su Y. (2016). Interferon-gamma-induced p27kip1 binds to and targets myc for proteasome-mediated degradation. Oncotarget.

[126] Malumbres M., Barbacid M. (2009). Cell cycle, cdks and cancer: A changing paradigm. Nat. Rev. Cancer.

[127] Ishimi Y., Komamura-Kohno Y., You Z., Omori A., Kitagawa M. (2000). Inhibition of mcm4,6,7 helicase activity by phosphorylation with cyclin a/cdk2. J. Biol. Chem..

[128] Jiang W., Wells N.J., Hunter T. (1999). Multistep regulation of DNA replication by cdk phosphorylation of hscdc6. Proc. Natl. Acad. Sci. USA.

[129] Li J., Deng M., Wei Q., Liu T., Tong X., Ye X. (2011). Phosphorylation of mcm3 protein by cyclin e/cyclin-dependent kinase 2 (cdk2) regulates its function in cell cycle. J. Biol. Chem..

[130] Petersen B.O., Lukas J., Sorensen C.S., Bartek J., Helin K. (1999). Phosphorylation of mammalian cdc6 by cyclin a/cdk2 regulates its subcellular localization. EMBO J..

[131] Geng Y., Yu Q., Sicinska E., Das M., Schneider J.E., Bhattacharya S., Rideout W.M., Bronson R.T., Gardner H., Sicinski P. (2003). Cyclin e ablation in the mouse. Cell.

[132] Chuang L.C., Teixeira L.K., Wohlschlegel J.A., Henze M., Yates J.R., Mendez J., Reed S.I. (2009). Phosphorylation of mcm2 by cdc7 promotes pre-replication complex assembly during cell-cycle re-entry. Mol. Cell.

[133] Dominguez-Sola D., Ying C.Y., Grandori C., Ruggiero L., Chen B., Li M., Galloway D.A., Gu W., Gautier J., Dalla-Favera R. (2007). Non-transcriptional control of DNA replication by c-myc. Nature.

[134] Campaner S., Doni M., Hydbring P., Verrecchia A., Bianchi L., Sardella D., Schleker T., Perna D., Tronnersjo S., Murga M. (2010). Cdk2 suppresses cellular senescence induced by the c-myc oncogene. Nat. Cell Biol..

[135] Felsher D.W., Bishop J.M. (1999). Transient excess of myc activity can elicit genomic instability and tumorigenesis. Proc. Natl. Acad. Sci. USA.

[136] Li Q., Dang C.V. (1999). C-myc overexpression uncouples DNA replication from mitosis. Mol. Cell. Biol..

[137] Maya-Mendoza A., Ostrakova J., Kosar M., Hall A., Duskova P., Mistrik M., Merchut-Maya J.M., Hodny Z., Bartkova J., Christensen C. (2015). Myc and ras oncogenes engage different energy metabolism programs and evoke distinct patterns of oxidative and DNA replication stress. Mol. Oncol..

[138] Reimann M., Loddenkemper C., Rudolph C., Schildhauer I., Teichmann B., Stein H., Schlegelberger B., Dorken B., Schmitt C.A. (2007). The myc-evoked DNA damage response accounts for treatment resistance in primary lymphomas in vivo. Blood.

[139] Robinson K., Asawachaicharn N., Galloway D.A., Grandori C. (2009). C-myc accelerates s-phase and requires wrn to avoid replication stress. PLoS ONE.

[140] Vafa O., Wade M., Kern S., Beeche M., Pandita T.K., Hampton G.M., Wahl G.M. (2002). C-myc can induce DNA damage, increase reactive oxygen species, and mitigate p53 function: A mechanism for oncogene-induced genetic instability. Mol. Cell.

[141] Branzei D., Foiani M. (2008). Regulation of DNA repair throughout the cell cycle. Nat. Rev. Mol. Cell Biol..

[142] Buis J., Stoneham T., Spehalski E., Ferguson D.O. (2012). Mre11 regulates ctip-dependent double-strand break repair by interaction with cdk2. Nat. Struct. Mol. Biol..

[143] Deans A.J., Khanna K.K., McNees C.J., Mercurio C., Heierhorst J., McArthur G.A. (2006). Cyclin-dependent kinase 2 functions in normal DNA repair and is a therapeutic target in brca1-deficient cancers. Cancer Res..

[144] Huang H., Regan K.M., Lou Z., Chen J., Tindall D.J. (2006). Cdk2-dependent phosphorylation of foxo1 as an apoptotic response to DNA damage. Science.

[145] Huertas P., Jackson S.P. (2009). Human ctip mediates cell cycle control of DNA end resection and double strand break repair. J. Biol. Chem..

[146] Polato F., Callen E., Wong N., Faryabi R., Bunting S., Chen H.T., Kozak M., Kruhlak M.J., Reczek C.R., Lee W.H. (2014). Ctip-mediated resection is essential for viability and can operate independently of brca1. J. Exp. Med..

[147] Yu X., Chen J. (2004). DNA damage-induced cell cycle checkpoint control requires ctip, a phosphorylation-dependent binding partner of brca1 c-terminal domains. Mol. Cell. Biol..

[148] Kuilman T., Michaloglou C., Mooi W.J., Peeper D.S. (2010). The essence of senescence. Genes Dev..

[149] Larsson L.G. (2011). Oncogene- and tumor suppressor gene-mediated suppression of cellular senescence. Semin. Cancer Biol..

[150] Munoz-Espin D., Serrano M. (2014). Cellular senescence: From physiology to pathology. Nat. Rev. Mol. Cell Biol..

[151] Luoto K.R., Meng A.X., Wasylishen A.R., Zhao H., Coackley C.L., Penn L.Z., Bristow R.G. (2010). Tumor cell kill by c-myc depletion: Role of myc-regulated genes that control DNA double-strand break repair. Cancer Res..

[152] Guerra L., Albihn A., Tronnersjo S., Yan Q., Guidi R., Stenerlow B., Sterzenbach T., Josenhans C., Fox J.G., Schauer D.B. (2010). Myc is required for activation of the atm-dependent checkpoints in response to DNA damage. PLoS ONE.

[153] Sherr C.J., Roberts J.M. (1999). Cdk inhibitors: Positive and negative regulators of g1-phase progression. Genes Dev..

[154] Blain S.W., Montalvo E., Massague J. (1997). Differential interaction of the cyclin-dependent kinase (cdk) inhibitor p27kip1 with cyclin a-cdk2 and cyclin d2-cdk4. J. Biol. Chem..

[155] Polyak K., Lee M.H., Erdjument-Bromage H., Koff A., Roberts J.M., Tempst P., Massague J. (1994). Cloning of p27kip1, a cyclin-dependent kinase inhibitor and a potential mediator of extracellular antimitogenic signals. Cell.

[156] Polyak K., Kato J.Y., Solomon M.J., Sherr C.J., Massague J., Roberts J.M., Koff A. (1994). P27kip1, a cyclin-cdk inhibitor, links transforming growth factor-beta and contact inhibition to cell cycle arrest. Genes Dev..

[157] Toyoshima H., Hunter T. (1994). P27, a novel inhibitor of g1 cyclin-cdk protein kinase activity, is related to p21. Cell.

[158] Chu I.M., Hengst L., Slingerland J.M. (2008). The cdk inhibitor p27 in human cancer: Prognostic potential and relevance to anticancer therapy. Nat. Rev. Cancer.

[159] Lu Z., Hunter T. (2010). Ubiquitylation and proteasomal degradation of the p21(cip1), p27(kip1) and p57(kip2) cdk inhibitors. Cell Cycle.

[160] Slingerland J., Pagano M. (2000). Regulation of the cdk inhibitor p27 and its deregulation in cancer. J. Cell. Physiol..

[161] Carrano A.C., Eytan E., Hershko A., Pagano M. (1999). Skp2 is required for ubiquitin-mediated degradation of the cdk inhibitor p27. Nat. Cell Biol..

[162] Sutterluty H., Chatelain E., Marti A., Wirbelauer C., Senften M., Muller U., Krek W. (1999). P45skp2 promotes p27kip1 degradation and induces s phase in quiescent cells. Nat. Cell Biol..

[163] Zhang H., Kobayashi R., Galaktionov K., Beach D. (1995). P19skp1 and p45skp2 are essential elements of the cyclin a-cdk2 s phase kinase. Cell.

[164] Lisztwan J., Marti A., Sutterluty H., Gstaiger M., Wirbelauer C., Krek W. (1998). Association of human cul-1 and ubiquitin-conjugating enzyme cdc34 with the f-box protein p45(skp2): Evidence for evolutionary conservation in the subunit composition of the cdc34-scf pathway. EMBO J..

[165] Lyapina S.A., Correll C.C., Kipreos E.T., Deshaies R.J. (1998). Human cul1 forms an evolutionarily conserved ubiquitin ligase complex (scf) with skp1 and an f-box protein. Proc. Natl. Acad. Sci. USA.

[166] Yu Z.K., Gervais J.L., Zhang H. (1998). Human cul-1 associates with the skp1/skp2 complex and regulates p21(cip1/waf1) and cyclin d proteins. Proc. Natl. Acad. Sci. USA.

[167] Frescas D., Pagano M. (2008). Deregulated proteolysis by the f-box proteins skp2 and beta-trcp: Tipping the scales of cancer. Nat. Rev. Cancer.

[168] Huang H., Regan K.M., Wang F., Wang D., Smith D.I., van Deursen J.M., Tindall D.J. (2005). Skp2 inhibits foxo1 in tumor suppression through ubiquitin-mediated degradation. Proc. Natl. Acad. Sci. USA.

[169] Lee S.W., Li C.F., Jin G., Cai Z., Han F., Chan C.H., Yang W.L., Li B.K., Rezaeian A.H., Li H.Y. (2015). Skp2-dependent ubiquitination and activation of lkb1 is essential for cancer cell survival under energy stress. Mol. Cell.

[170] Jin G., Lee S.W., Zhang X., Cai Z., Gao Y., Chou P.C., Rezaeian A.H., Han F., Wang C.Y., Yao J.C. (2015). Skp2-mediated raga ubiquitination elicits a negative feedback to prevent amino-acid-dependent mtorc1 hyperactivation by recruiting gator1. Mol. Cell.

[171] Wu J., Zhang X., Zhang L., Wu C.Y., Rezaeian A.H., Chan C.H., Li J.M., Wang J., Gao Y., Han F. (2012). Skp2 e3 ligase integrates atm activation and homologous recombination repair by ubiquitinating nbs1. Mol. Cell.

[172] Zhao H., Bauzon F., Fu H., Lu Z., Cui J., Nakayama K., Nakayama K.I., Locker J., Zhu L. (2013). Skp2 deletion unmasks a p27 safeguard that blocks tumorigenesis in the absence of prb and p53 tumor suppressors. Cancer Cell.

[173] Nakayama K., Nagahama H., Minamishima Y.A., Miyake S., Ishida N., Hatakeyama S., Kitagawa M., Iemura S., Natsume T., Nakayama K.I. (2004). Skp2-mediated degradation of p27 regulates progression into mitosis. Dev. Cell.

[174] Kossatz U., Dietrich N., Zender L., Buer J., Manns M.P., Malek N.P. (2004). Skp2-dependent degradation of p27kip1 is essential for cell cycle progression. Genes Dev..

[175] Nakayama K., Nagahama H., Minamishima Y.A., Matsumoto M., Nakamichi I., Kitagawa K., Shirane M., Tsunematsu R., Tsukiyama T., Ishida N. (2000). Targeted disruption of skp2 results in accumulation of cyclin e and p27(kip1), polyploidy and centrosome overduplication. EMBO J..

[176] Aleem E., Kiyokawa H., Kaldis P. (2005). Cdc2-cyclin e complexes regulate the g1/s phase transition. Nat. Cell Biol..

[177] Signoretti S., Di Marcotullio L., Richardson A., Ramaswamy S., Isaac B., Rue M., Monti F., Loda M., Pagano M. (2002). Oncogenic role of the ubiquitin ligase subunit skp2 in human breast cancer. J. Clin. Investig..

[178] Gstaiger M., Jordan R., Lim M., Catzavelos C., Mestan J., Slingerland J., Krek W. (2001). Skp2 is oncogenic and overexpressed in human cancers. Proc. Natl. Acad. Sci. USA.

[179] Chiappetta G., De Marco C., Quintiero A., Califano D., Gherardi S., Malanga D., Scrima M., Montero-Conde C., Cito L., Monaco M. (2007). Overexpression of the s-phase kinase-associated protein 2 in thyroid cancer. Endocr. Relat. Cancer.

[180] Slotky M., Shapira M., Ben-Izhak O., Linn S., Futerman B., Tsalic M., Hershko D.D. (2005). The expression of the ubiquitin ligase subunit cks1 in human breast cancer. Breast Cancer Res..

[181] Ganoth D., Bornstein G., Ko T.K., Larsen B., Tyers M., Pagano M., Hershko A. (2001). The cell-cycle regulatory protein cks1 is required for scf(skp2)-mediated ubiquitinylation of p27. Nat. Cell Biol..

[182] Spruck C., Strohmaier H., Watson M., Smith A.P., Ryan A., Krek T.W., Reed S.I. (2001). A cdk-independent function of mammalian cks1: Targeting of scf(skp2) to the cdk inhibitor p27kip1. Mol. Cell.

[183] Bondar T., Kalinina A., Khair L., Kopanja D., Nag A., Bagchi S., Raychaudhuri P. (2006). Cul4a and ddb1 associate with skp2 to target p27kip1 for proteolysis involving the cop9 signalosome. Mol. Cell. Biol..

[184] Vernell R., Helin K., Muller H. (2003). Identification of target genes of the p16ink4a-prb-e2f pathway. J. Biol. Chem..

[185] Zhang L., Wang C. (2006). F-box protein skp2: A novel transcriptional target of e2f. Oncogene.

[186] Wei W., Ayad N.G., Wan Y., Zhang G.J., Kirschner M.W., Kaelin W.G. (2004). Degradation of the scf component skp2 in cell-cycle phase g1 by the anaphase-promoting complex. Nature.

[187] Binne U.K., Classon M.K., Dick F.A., Wei W., Rape M., Kaelin W.G., Naar A.M., Dyson N.J. (2007). Retinoblastoma protein and anaphase-promoting complex physically interact and functionally cooperate during cell-cycle exit. Nat. Cell Biol..

[188] Ji P., Jiang H., Rekhtman K., Bloom J., Ichetovkin M., Pagano M., Zhu L. (2004). An rb-skp2-p27 pathway mediates acute cell cycle inhibition by rb and is retained in a partial-penetrance rb mutant. Mol. Cell.

[189] Jamal A., Swarnalatha M., Sultana S., Joshi P., Panda S.K., Kumar V. (2015). The g1 phase e3 ubiquitin ligase truss that gets deregulated in human cancers is a novel substrate of the s-phase e3 ubiquitin ligase skp2. Cell Cycle.

[190] Von der Lehr N., Johansson S., Wu S., Bahram F., Castell A., Cetinkaya C., Hydbring P., Weidung I., Nakayama K., Nakayama K.I. (2003). The f-box protein skp2 participates in c-myc proteosomal degradation and acts as a cofactor for c-myc-regulated transcription. Mol. Cell.

[191] Kim S.Y., Herbst A., Tworkowski K.A., Salghetti S.E., Tansey W.P. (2003). Skp2 regulates myc protein stability and activity. Mol. Cell.

[192] Adler A.S., Lin M., Horlings H., Nuyten D.S., van de Vijver M.J., Chang H.Y. (2006). Genetic regulators of large-scale transcriptional signatures in cancer. Nat. Genet..

[193] Fujii M., Lyakh L.A., Bracken C.P., Fukuoka J., Hayakawa M., Tsukiyama T., Soll S.J., Harris M., Rocha S., Roche K.C. (2006). Snip1 is a candidate modifier of the transcriptional activity of c-myc on e box-dependent target genes. Mol. Cell.

[194] Chan C.H., Lee S.W., Wang J., Lin H.K. (2010). Regulation of skp2 expression and activity and its role in cancer progression. ScientificWorldJournal.

[195] Zhang B., Ji L.H., Liu W., Zhao G., Wu Z.Y. (2013). Skp2-rnai suppresses proliferation and migration of gallbladder carcinoma cells by enhancing p27 expression. World J. Gastroenterol..

[196] Zhuang D., Mannava S., Grachtchouk V., Tang W.H., Patil S., Wawrzyniak J.A., Berman A.E., Giordano T.J., Prochownik E.V., Soengas M.S. (2008). C-myc overexpression is required for continuous suppression of oncogene-induced senescence in melanoma cells. Oncogene.

[197] Lin H.K., Chen Z., Wang G., Nardella C., Lee S.W., Chan C.H., Yang W.L., Wang J., Egia A., Nakayama K.I. (2010). Skp2 targeting suppresses tumorigenesis by arf-p53-independent cellular senescence. Nature.

[198] Zhang Q., Spears E., Boone D.N., Li Z., Gregory M.A., Hann S.R. (2013). Domain-specific c-myc ubiquitylation controls c-myc transcriptional and apoptotic activity. Proc. Natl. Acad. Sci. USA.

[199] Larsson L.G. (2006). Snip1: Myc’s new helper in transcriptional activation. Mol. Cell.

[200] Thomas D., Tyers M. (2000). Transcriptional regulation: Kamikaze activators. Curr. Biol..

[201] Lipford J.R., Deshaies R.J. (2003). Diverse roles for ubiquitin-dependent proteolysis in transcriptional activation. Nat. Cell Biol..

[202] Muratani M., Tansey W.P. (2003). How the ubiquitin-proteasome system controls transcription. Nat. Rev. Mol. Cell Biol..

[203] von der Lehr N., Johansson S., Larsson L.G. (2003). Implication of the ubiquitin/proteasome system in myc-regulated transcription. Cell Cycle.

[204] Geng F., Wenzel S., Tansey W.P. (2012). Ubiquitin and proteasomes in transcription. Annu. Rev. Biochem..

[205] Salghetti S.E., Muratani M., Wijnen H., Futcher B., Tansey W.P. (2000). Functional overlap of sequences that activate transcription and signal ubiquitin-mediated proteolysis. Proc. Natl. Acad. Sci. USA.

[206] Collins G.A., Goldberg A.L. (2017). The logic of the 26s proteasome. Cell.

[207] Zhao X., Heng J.I., Guardavaccaro D., Jiang R., Pagano M., Guillemot F., Iavarone A., Lasorella A. (2008). The hect-domain ubiquitin ligase huwe1 controls neural differentiation and proliferation by destabilizing the n-myc oncoprotein. Nat. Cell Biol..

[208] Kurosu T., Peterlin B.M. (2004). Vp16 and ubiquitin; binding of p-tefb via its activation domain and ubiquitin facilitates elongation of transcription of target genes. Curr. Biol..

[209] Barboric M., Zhang F., Besenicar M., Plemenitas A., Peterlin B.M. (2005). Ubiquitylation of cdk9 by skp2 facilitates optimal tat transactivation. J. Virol..

[210] Ferdous A., Gonzalez F., Sun L., Kodadek T., Johnston S.A. (2001). The 19s regulatory particle of the proteasome is required for efficient transcription elongation by rna polymerase ii. Mol. Cell.

[211] Gonzalez F., Delahodde A., Kodadek T., Johnston S.A. (2002). Recruitment of a 19s proteasome subcomplex to an activated promoter. Science.

[212] Chaves S., Baskerville C., Yu V., Reed S.I. (2010). Cks1, cdk1, and the 19s proteasome collaborate to regulate gene induction-dependent nucleosome eviction in yeast. Mol. Cell. Biol..

[213] Morris M.C., Kaiser P., Rudyak S., Baskerville C., Watson M.H., Reed S.I. (2003). Cks1-dependent proteasome recruitment and activation of cdc20 transcription in budding yeast. Nature.

[214] Lee D., Ezhkova E., Li B., Pattenden S.G., Tansey W.P., Workman J.L. (2005). The proteasome regulatory particle alters the saga coactivator to enhance its interactions with transcriptional activators. Cell.

[215] Malik S., Shukla A., Sen P., Bhaumik S.R. (2009). The 19s proteasome subcomplex establishes a specific protein interaction network at the promoter for stimulated transcriptional initiation in vivo. J. Biol. Chem..

[216] Arabi A., Wu S., Ridderstrale K., Bierhoff H., Shiue C., Fatyol K., Fahlen S., Hydbring P., Soderberg O., Grummt I. (2005). C-myc associates with ribosomal DNA and activates rna polymerase i transcription. Nat. Cell Biol..

[217] Reid G., Hubner M.R., Metivier R., Brand H., Denger S., Manu D., Beaudouin J., Ellenberg J., Gannon F. (2003). Cyclic, proteasome-mediated turnover of unliganded and liganded eralpha on responsive promoters is an integral feature of estrogen signaling. Mol. Cell.

[218] Zhang H., Sun L., Liang J., Yu W., Zhang Y., Wang Y., Chen Y., Li R., Sun X., Shang Y. (2006). The catalytic subunit of the proteasome is engaged in the entire process of estrogen receptor-regulated transcription. EMBO J..

[219] Ostendorff H.P., Peirano R.I., Peters M.A., Schluter A., Bossenz M., Scheffner M., Bach I. (2002). Ubiquitination-dependent cofactor exchange on lim homeodomain transcription factors. Nature.

[220] Perissi V., Aggarwal A., Glass C.K., Rose D.W., Rosenfeld M.G. (2004). A corepressor/coactivator exchange complex required for transcriptional activation by nuclear receptors and other regulated transcription factors. Cell.

[221] Wu R.C., Feng Q., Lonard D.M., O’Malley B.W. (2007). Src-3 coactivator functional lifetime is regulated by a phospho-dependent ubiquitin time clock. Cell.

[222] Howard G.C., Tansey W.P. (2016). Interaction of gcn4 with target gene chromatin is modulated by proteasome function. Mol. Biol. Cell.

[223] Bouchard C., Dittrich O., Kiermaier A., Dohmann K., Menkel A., Eilers M., Luscher B. (2001). Regulation of cyclin d2 gene expression by the myc/max/mad network: Myc-dependent trrap recruitment and histone acetylation at the cyclin d2 promoter. Genes Dev..

[224] Amente S., Bertoni A., Morano A., Lania L., Avvedimento E.V., Majello B. (2010). Lsd1-mediated demethylation of histone h3 lysine 4 triggers myc-induced transcription. Oncogene.

[225] Lu W., Liu S., Li B., Xie Y., Adhiambo C., Yang Q., Ballard B.R., Nakayama K.I., Matusik R.J., Chen Z. (2015). Skp2 inactivation suppresses prostate tumorigenesis by mediating jarid1b ubiquitination. Oncotarget.

[226] Secombe J., Li L., Carlos L., Eisenman R.N. (2007). The trithorax group protein lid is a trimethyl histone h3k4 demethylase required for dmyc-induced cell growth. Genes Dev..

[227] Xu D., Li C.F., Zhang X., Gong Z., Chan C.H., Lee S.W., Jin G., Rezaeian A.H., Han F., Wang J. (2015). Skp2-macroh2a1-cdk8 axis orchestrates g2/m transition and tumorigenesis. Nat. Commun..

[228] Adams P.D. (2007). Remodeling of chromatin structure in senescent cells and its potential impact on tumor suppression and aging. Gene.

[229] Chandramohan V., Mineva N.D., Burke B., Jeay S., Wu M., Shen J., Yang W., Hann S.R., Sonenshein G.E. (2008). C-myc represses foxo3a-mediated transcription of the gene encoding the p27(kip1) cyclin dependent kinase inhibitor. J. Cell. Biochem..

[230] Fujita N., Sato S., Tsuruo T. (2003). Phosphorylation of p27kip1 at threonine 198 by p90 ribosomal protein s6 kinases promotes its binding to 14-3-3 and cytoplasmic localization. J. Biol. Chem..

[231] Liang J., Zubovitz J., Petrocelli T., Kotchetkov R., Connor M.K., Han K., Lee J.H., Ciarallo S., Catzavelos C., Beniston R. (2002). Pkb/akt phosphorylates p27, impairs nuclear import of p27 and opposes p27-mediated g1 arrest. Nat. Med..

[232] Morishita D., Katayama R., Sekimizu K., Tsuruo T., Fujita N. (2008). Pim kinases promote cell cycle progression by phosphorylating and down-regulating p27kip1 at the transcriptional and posttranscriptional levels. Cancer Res..

[233] Cowling V.H., Chandriani S., Whitfield M.L., Cole M.D. (2006). A conserved myc protein domain, mbiv, regulates DNA binding, apoptosis, transformation, and g2 arrest. Mol. Cell. Biol..

[234] Vervoorts J., Luscher-Firzlaff J., Luscher B. (2006). The ins and outs of myc regulation by posttranslational mechanisms. J. Biol. Chem..

[235] Wasylishen A.R., Chan-Seng-Yue M., Bros C., Dingar D., Tu W.B., Kalkat M., Chan P.K., Mullen P.J., Huang L., Meyer N. (2013). Myc phosphorylation at novel regulatory regions suppresses transforming activity. Cancer Res..

[236] Wasylishen A.R., Kalkat M., Kim S.S., Pandyra A., Chan P.K., Oliveri S., Sedivy E., Konforte D., Bros C., Raught B. (2014). Myc activity is negatively regulated by a c-terminal lysine cluster. Oncogene.

[237] Zhu L., Chiao C.Y., Enzer K.G., Stankiewicz A.J., Faller D.V., Dai Y. (2015). Sirt1 inactivation evokes antitumor activities in nsclc through the tumor suppressor p27. Mol. Cancer Res..

[238] Luscher B., Kuenzel E.A., Krebs E.G., Eisenman R.N. (1989). Myc oncoproteins are phosphorylated by casein kinase ii. EMBO J..

[239] Hauck L., Harms C., An J., Rohne J., Gertz K., Dietz R., Endres M., von Harsdorf R. (2008). Protein kinase ck2 links extracellular growth factor signaling with the control of p27(kip1) stability in the heart. Nat. Med..

[240] Pippa R., Espinosa L., Gundem G., Garcia-Escudero R., Dominguez A., Orlando S., Gallastegui E., Saiz C., Besson A., Pujol M.J. (2012). P27kip1 represses transcription by direct interaction with p130/e2f4 at the promoters of target genes. Oncogene.

[241] McArthur G.A., Foley K.P., Fero M.L., Walkley C.R., Deans A.J., Roberts J.M., Eisenman R.N. (2002). Mad1 and p27(kip1) cooperate to promote terminal differentiation of granulocytes and to inhibit myc expression and cyclin e-cdk2 activity. Mol. Cell. Biol..

[242] Hwang H.C., Martins C.P., Bronkhorst Y., Randel E., Berns A., Fero M., Clurman B.E. (2002). Identification of oncogenes collaborating with p27kip1 loss by insertional mutagenesis and high-throughput insertion site analysis. Proc. Natl. Acad. Sci. USA.

[243] Seviour E.G., Sehgal V., Lu Y., Luo Z., Moss T., Zhang F., Hill S.M., Liu W., Maiti S.N., Cooper L. (2016). Functional proteomics identifies mirnas to target a p27/myc/phospho-rb signature in breast and ovarian cancer. Oncogene.

[244] Caraballo J.M., Acosta J.C., Cortes M.A., Albajar M., Gomez-Casares M.T., Batlle-Lopez A., Cuadrado M.A., Onaindia A., Bretones G., Llorca J. (2014). High p27 protein levels in chronic lymphocytic leukemia are associated to low myc and skp2 expression, confer resistance to apoptosis and antagonize myc effects on cell cycle. Oncotarget.

[245] Kim S.S., Meitner P., Konkin T.A., Cho Y.S., Resnick M.B., Moss S.F. (2006). Altered expression of skp2, c-myc and p27 proteins but not mrna after h. Pylori eradication in chronic gastritis. Mod. Pathol..

[246] Besson A., Assoian R.K., Roberts J.M. (2004). Regulation of the cytoskeleton: An oncogenic function for cdk inhibitors?. Nat. Rev. Cancer.

[247] Denicourt C., Saenz C.C., Datnow B., Cui X.S., Dowdy S.F. (2007). Relocalized p27kip1 tumor suppressor functions as a cytoplasmic metastatic oncogene in melanoma. Cancer Res..

[248] Asghar U., Witkiewicz A.K., Turner N.C., Knudsen E.S. (2015). The history and future of targeting cyclin-dependent kinases in cancer therapy. Nat. Rev. Drug Discov..

[249] Goga A., Yang D., Tward A.D., Morgan D.O., Bishop J.M. (2007). Inhibition of cdk1 as a potential therapy for tumors over-expressing myc. Nat. Med..

[250] Molenaar J.J., Ebus M.E., Geerts D., Koster J., Lamers F., Valentijn L.J., Westerhout E.M., Versteeg R., Caron H.N. (2009). Inactivation of cdk2 is synthetically lethal to mycn over-expressing cancer cells. Proc. Natl. Acad. Sci. USA.

[251] Hydbring P., Wang Y., Fassl A., Li X., Matia V., Otto T., Choi Y.J., Sweeney K.E., Suski J.M., Yin H. (2017). Cell-cycle-targeting micrornas as therapeutic tools against refractory cancers. Cancer Cell.

[252] Schiewer M.J., Knudsen K.E. (2017). Not so fast: Cultivating mirs as kinks in the chain of the cell cycle. Cancer Cell.

[253] Janghorban M., Farrell A.S., Allen-Petersen B.L., Pelz C., Daniel C.J., Oddo J., Langer E.M., Christensen D.J., Sears R.C. (2014). Targeting c-myc by antagonizing pp2a inhibitors in breast cancer. Proc. Natl. Acad. Sci. USA.

[254] Wu L., Grigoryan A.V., Li Y., Hao B., Pagano M., Cardozo T.J. (2012). Specific small molecule inhibitors of skp2-mediated p27 degradation. Chem. Biol..

[255] Ooi L.C., Watanabe N., Futamura Y., Sulaiman S.F., Darah I., Osada H. (2013). Identification of small molecule inhibitors of p27(kip1) ubiquitination by high-throughput screening. Cancer Sci..

[256] Pavlides S.C., Huang K.T., Reid D.A., Wu L., Blank S.V., Mittal K., Guo L., Rothenberg E., Rueda B., Cardozo T. (2013). Inhibitors of scf-skp2/cks1 e3 ligase block estrogen-induced growth stimulation and degradation of nuclear p27kip1: Therapeutic potential for endometrial cancer. Endocrinology.

[257] Ludwik K.A., Lannigan D.A. (2016). Ribosomal s6 kinase (rsk) modulators: A patent review. Expert Opin. Ther. Pat..

[258] Pretre V., Wicki A. (2017). Inhibition of akt and other agc kinases: A target for clinical cancer therapy?. Semin. Cancer Biol..

[259] McKeown M.R., Bradner J.E. (2014). Therapeutic strategies to inhibit myc. Cold Spring Harb. Perspect. Med..

[260] Fletcher S., Prochownik E.V. (2015). Small-molecule inhibitors of the myc oncoprotein. Biochim. Biophys. Acta.

[261] Prochownik E.V., Vogt P.K. (2010). Therapeutic targeting of myc. Genes Cancer.

[262] Cetinkaya C., Hultquist A., Su Y., Wu S., Bahram F., Pahlman S., Guzhova I., Larsson L.G. (2007). Combined ifn-gamma and retinoic acid treatment targets the n-myc/max/mad1 network resulting in repression of n-myc target genes in mycn-amplified neuroblastoma cells. Mol. Cancer Ther..

[263] Miller C.H., Maher S.G., Young H.A. (2009). Clinical use of interferon-gamma. Ann. N. Y. Acad. Sci..

[264] Zaidi M.R., Merlino G. (2011). The two faces of interferon-gamma in cancer. Clin. Cancer Res..

[265] Finn O.J. (2008). Cancer immunology. N. Engl. J. Med..

[266] Braumuller H., Wieder T., Brenner E., Assmann S., Hahn M., Alkhaled M., Schilbach K., Essmann F., Kneilling M., Griessinger C. (2013). T-helper-1-cell cytokines drive cancer into senescence. Nature.

[267] Rakhra K., Bachireddy P., Zabuawala T., Zeiser R., Xu L., Kopelman A., Fan A.C., Yang Q., Braunstein L., Crosby E. (2010). Cd4(+) T cells contribute to the remodeling of the microenvironment required for sustained tumor regression upon oncogene inactivation. Cancer Cell.

[268] Mellman I., Coukos G., Dranoff G. (2011). Cancer immunotherapy comes of age. Nature.

[269] Huang B., Abraham W.D., Zheng Y., Bustamante Lopez S.C., Luo S.S., Irvine D.J. (2015). Active targeting of chemotherapy to disseminated tumors using nanoparticle-carrying t cells. Sci. Transl. Med..

